# Thiosemicarbazone Structures Including Nickelophilic Interaction as Well as Both Hydrogen Bonding and π–π Stacking Interactions: NLO, Electrochemical, Chromism, and Spectroelectrochemical Properties

**DOI:** 10.1021/acsomega.5c04784

**Published:** 2025-10-14

**Authors:** Elif Avcu Altıparmak, Özlem Uğuz Neli, Tülay Bal-Demirci, Namık Özdemir, Atıf Koca

**Affiliations:** † Department of Chemistry, Engineering Faculty, 64298 İstanbul University-Cerrahpaşa, 34320 İstanbul, Türkiye; ‡ Department of Chemistry, Stockholm University, 10691 Stockholm, Sweden; § Chemical Engineering Department, Engineering Faculty, 52982 Marmara University, 34722 Istanbul, Türkiye; ∥ Department of Physics, Faculty of Science, Ondokuz Mayıs University, 55139 Samsun, Türkiye

## Abstract

Nickelophilic complexes were formed from salicylaldehyde thiosemicarbazone and nitro-substituted aniline through second-sphere coordination interactions. The structures of the complexes were characterized by elemental analysis, IR, ^1^H NMR, UV–vis spectroscopy, and single-crystal X-ray diffraction. Additionally, the structural, spectroscopic, electronic, and solvatochromic properties were investigated, and the nonlinear optic properties were studied using density functional theory (DFT) calculations and diffuse reflectance spectroscopy. The energy gap values suggested that the complexes exhibited semiconductor-like behavior. X-ray crystallographic studies revealed that the two phenolato oxygen atoms and two azomethine nitrogen atoms of the doubly deprotonated ONNO tetradentate Schiff base occupy the corners of a square-planar geometry around the metal atom. The structures were found to be stabilized by hydrogen bonding and π–π stacking, in addition to Ni···Ni interactions. Electrochemical characterizations of the starting material and complexes were carried out to evaluate the influence of the 2-nitroaniline and 2,4-dinitroaniline coligands as well as the coordination of the starting material with the Ni­(II) cation, which exhibited very complex reduction processes with reduction responses significantly influenced by the applied waveforms of the voltammetric excitations. Additionally, complexation also influenced the in situ spectroelectrochemical responses of the starting material.

## Graphical abstract

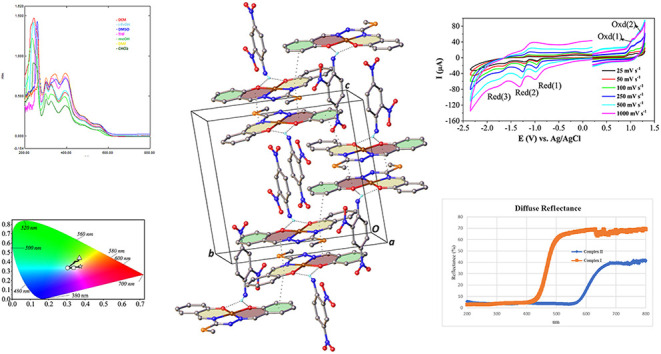

## 1 Introduction

Since Lehn was awarded the Nobel Prize for his work on supramolecular chemistry in 1987,^1,2^ the synthesis of new supramolecular structures has been the subject of extensive research^3^ and has led to unconventional new themes such as host–guest chemistry, nonlinear optical (NLO) properties, 3D-/4D-printing systems,^4^ gas adsorption,^5^ and gas storage and separation.^6^

Physical interactions, such as intra- or intermolecular hydrogen bonding, π–π stacking of arenes, van der Waals forces, electrostatic interactions, and metal–metal contacts, are considered highly significant, as they contribute to the formation of supramolecular and polymer structures.^7^ Another type of physical interaction is metal–metal contact, known as metallophilic interactions. Metallophilic interactions occur between closed-shell (d^10^) and pseudoclosed-shell (d^8^) metal ions in transition metal or organometallic complexes.^8–10^

The term “metallophilic” is general nomenclature derived from a combination of the words “metal” in the Latin language and “philein” (having a distinct preference, love) in the Greek language, with examples including auriophilic, mercurophilic, argentophilic, and nickelophilic.^11^ Among these, nickelophilic interactions, i.e., Ni···Ni contacts, are relatively uncommon. These rare metallophilic interactions are not covalent bonds but rather very strong van der Waals forces. Their interaction energies are approximately close to hydrogen bonding.^12^ Eleven nickelophilic complexes showing Ni···Ni interactions in the ONNO coordination mode were found in the Cambridge Structural Database (CSD). The Ni···Ni interaction distances (in Å) in these complexes range from 3.251 to 3.419 Å (Table S1).

The first comprehensive article on metal–metal bonding was published by Coffey, Lewis, and Nyholm in 1964.^13^ Metallophilic interactions affect physical and structural properties such as luminescence, conductivity, catalysis, and nonlinear optical (NLO) properties of compounds.

In general, typical NLO candidate compounds include π-conjugated bridges and strong donor and acceptor groups. Increasing the conjugation within the molecule and introducing donor and acceptor groups enhances nonlinear optical properties.^14–16^ Nonlinear optical properties (NLO) have an important place in many areas. Optical materials featuring NLO characteristics are extensively studied for applications in electrooptics, telecommunications, data acquisition and retrieval, computing, and display technology.

Thiosemicarbazones that contain more than one donor atom (N, S, and additional donor atoms) are a class of ligands that have attracted significant attention in coordination chemistry due to their ability to engage in strong physical interactions and exhibit variable coordination modes.^15–20^ Many applications of thiosemicarbazones, such as cancer treatment,^21–23^ sensors,^24^ catalytic applications,^25–27^ corrosion inhibition,^28–30^ and energy conversion and storage devices,^31,32^ are related to their electrochemical functionalities, which can be tailored by modifying the complexation of metal cations and their substituents. ONS-donor thiosemicarbazone derivatives generally exhibit a successive two-electron (2e^–^) or sequential one-electron (1e^–^) imine (–N=CH–)-based reduction. The imine reduction of these ligands results in hydrazo products, while further reduction via a two-electron process leads to amine products.^33^ For instance, in our previous paper, nickel­(II) complexes of 5-chloro-2-hydroxybenzophenone-N-R-thiosemicarbazone exhibited 2e^–^ reduction waves at more negative potentials, in addition to Ni^II^/Ni^III^ oxidation.^34^ In another study, R. Prabhakaran reported a Ni^II^/Ni^III^ oxidation couple and a ligand-based reduction at −1.65 V for a nickel­(II) thiosemicarbazone complex.^35^ El-Shazly and co-workers investigated the electrochemistry of several Ni­(II) complexes with thiosemicarbazone derivatives and reported both Ni­(II)/Ni­(I) and Ni­(III)/Ni­(II) couples, without any ligand-based processes.^36^ In contrast, Huseynova and co-workers reported Ni^II^/Ni^I^ and Ni^III^/Ni^II^ couples along with irreversible ligand-based oxidation and reduction waves at 1.22, −0.68, 0.88, and −1.35 V versus SCE for the thiosemicarbazone of the glyoxylic acid (H_2_GAT) complex of nickel.^37^ Studies in the literature indicate that the redox behavior of the ONS-donor thiosemicarbazone derivatives generally varies with the type of coordinated metal cations and substituent environments. The electrolyte of the electrochemical analysis also influences the redox mechanism of these complexes. To assess the potential functionality of newly synthesized moieties, a detailed electrochemical analysis should be performed.

This paper presents the synthesis and properties of stabilized nickelophilic thiosemicarbazone complexes via a second-sphere coordination interaction with a nitro-substituted aniline compound (Scheme 1). The structures were characterized by spectroscopic methods, and their crystal structures were determined by single-crystal X-ray crystallography. The stabilization of these structures was achieved not only through Ni···Ni interactions but also via hydrogen bonding and π–π stacking (Scheme 2). The detailed electrochemical responses of the starting material and complexes are reported to evaluate their potential use in various electrochemical applications. Density functional theory (DFT) calculations were performed to study the structural, spectroscopic, and electronic properties of the complexes, including frontier molecular orbitals and NLO properties. The band gaps of the synthesized complexes were determined using diffuse reflectance spectra. Additionally, the solvatochromic properties of the complexes were investigated in commonly used solvents, including dimethyl sulfoxide (DMSO), dimethylformamide (DMF), isopropyl alcohol (*i*-PrOH), methanol (MeOH), tetrahydrofuran (THF), dichloromethane (DCM), and chloroform (CHCl_3_).

**Scheme 1:**
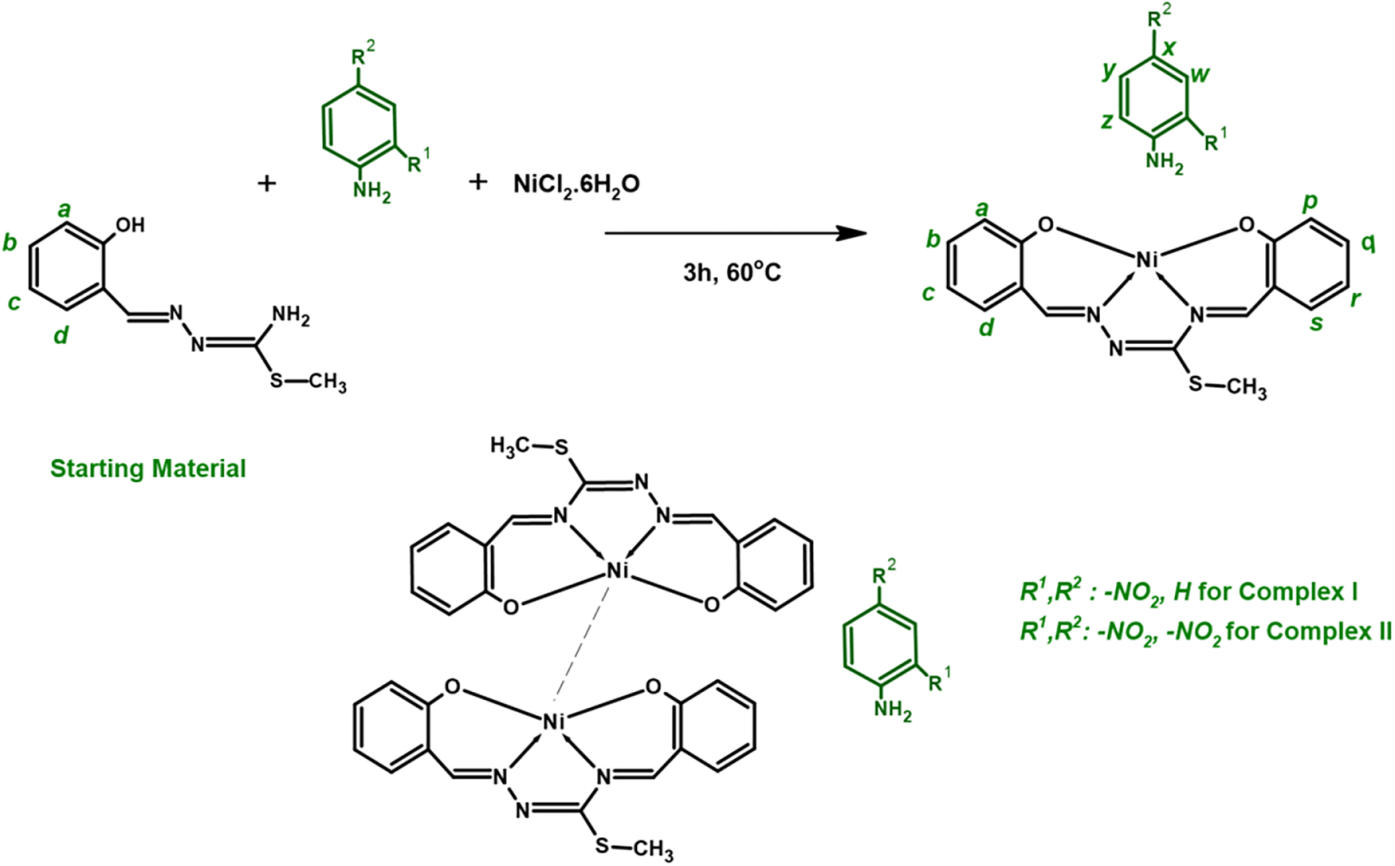
Formation of the Nickelophilic Complexes

**Scheme 2:**
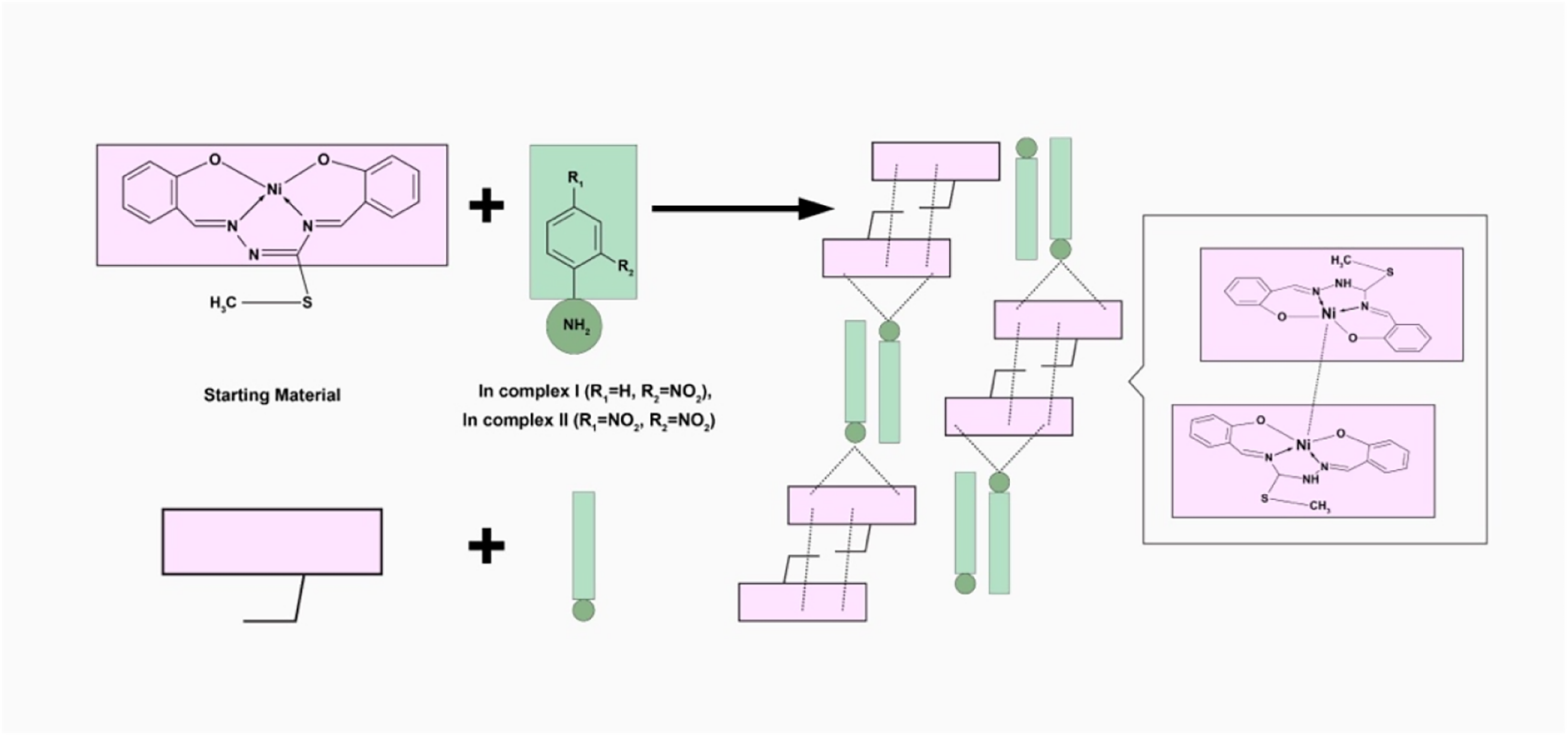
Schematic Visualization of Ni···Ni, Hydrogen Bonding and π–π Stacking Interactions in the Crystal Structures of Nickel­(II) Complexes, **Complex I** and **Complex II,** with an ONNO Coordination Mode^a^

a The 2,4-dinitroaniline compound is visualized in green, whereas the template nickel­(II) complex is shown in pink. The Ni···Ni interaction between the template complexes is highlighted in the bubble.

## 2 Experimental Section

### 2.1 Chemicals and Apparatus

All chemicals were of reagent grade and were used as received without further purification. Elemental analyses were performed using a Thermo Finnigan Flash EA 1112 Series Elemental Analyzer. IR spectra of the compounds were recorded on a Cary 630 FT-IR spectrometer with Diamond ATR from Agilent. The ^1^H NMR spectra were recorded in DMSO on a Bruker AVANCE-500 spectrometer. Ultraviolet–visible (UV–vis) spectra were obtained with a Shimadzu UV-2600 spectrophotometer using 5 × 10^–5^ M solutions in CHCl_3_. Optical and band gap measurements of the complexes were performed by using a Shimadzu 2600 UV–vis–NIR spectrophotometer. UV–vis diffuse and relative specular reflectance spectra were measured at room temperature with a Shimadzu-type 2600 UV spectrophotometer equipped with an ISR-2600 Plus two-detector integrating sphere covering the spectral range from 200 to 1400 nm.

### 2.2 Synthesis

#### 2.2.1 Starting Material: Salicylaldehyde-*S*-methylthiosemicarbazone

The starting material was prepared by the reaction of 2-hydroxybenzaldehyde and *S*-methylthiosemicarbazide according to the literature method in methanol. The resulting powdery yellow product was dried under vacuum.^38^

#### 2.2.2 Characterization Data of the Starting Material

Color: yellow; yield: 93%; mp (°C): 160–162; elemental analysis: Anal. Calcd for C_9_H_11_N_3_OS (209.27 g/mol): calculated: C, 51.65; H, 5.30; N, 20.08; S, 15.32; found: C, 51.63; H, 5.34; N, 20.09; S, 15.28%. IR (cm^–1^): ν­(–OH) 3517; ν_asym_(N–H) 3458; ν_sym_(N–H) 3278; δ­(N–H) 1635; ν­(C=N) 1617, 1602; ν­(C–O) 1148; ν­(C–S) 747. UV–vis (5 × 10^–5^ M, CHCl_3_) (λ (ε)): 346.5 (16890), 333.5 (21780), 304.5 (21120), 293 (21900), 239.5 (16420). ^1^H NMR (DMSO-*d*_6_, 25 °C, ppm): 11.58, 10.69 (s, *i*:1/2, 1H, OH); 8.45, 8.31 (s, 1H, *i*:1/2, CH=N^1^); 7.44 (d, 1H, *J* = 7.44, *d*); 7.19 (m, 1H, *b*); 6.92 (s, 2H, NH_2_); 6.88 (s, 2H, a,*c*); 2.39 (s, 3H, S-CH_3_).

#### 2.2.3 Ni­(II) Complexes of Thiosemicarbazone

##### 2.2.3.1 **Complex I** and **Complex II**

**Complex I** was prepared by the reaction of 2-hydroxy-benzaldehyde-*S*-methyl-isothiosemicarbazone, 2-nitroaniline, and NiCl_2_.6H_2_O. 2-Hydroxy-benzaldehyde-*S*-methyl-isothiosemicarbazone (0.21 g, 1 mmol), 2-hydroxybenzaldehyde (0.12 g, 1 mmol), and NiCl_2_.6H_2_O (0.23 g, 1 mmol) were stirred for 1 h in ethanol. A bright red solution was formed. After that, 2-nitroaniline (0.14 g, 1 mmol) was added to the mixture as a seconder ligand, and the color of the solution turned brownish red. The reaction mixture was refluxed for 3 h and filtered off. The brown-red powder product was recrystallized in ethanol several times until a single crystal was obtained and dried under vacuum. **Complex II** was synthesized in a similar way by using 2,4-dinitroaniline instead of 2-nitroaniline.

##### 2.2.3.2 Characterization Data of **Complex I**

Color: brownish red; yield: 47%; mp (°C): 182 (decomposition), 188 (melting); anal. calcd for C_22_H_19_N_5_NiO_4_S (508.19 g/mol): calculated: C, 52.00; H, 3.77; N, 13.78; S, 6.31%. Found: C, 52.02; H, 3.76; N, 13.80; S, 6.34%. IR (cm^–1^): ν_asym_(N–H) 3479, ν_sym_(N–H) 3302; ν­(C=CH) 3157, 2948, 2869; δ­(N–H) 1621; ν­(C=N) 1604, 1599, 1578; ν­(C–O) 1140, 1121; ν_asym_(N–O) 1543; ν_sym_(N–O) 1345; ν­(C–S) 697. UV–vis (5 × 10^–5^ M, CHCl_3_) (λ (ε)): 228 (11480), 240.5 (22140); 281 (8220), 300 (8540); 322.5 (6200); 395 (7700), 474 (2540), 549.5 (1460). ^1^H NMR (DMSO-*d*_6_, 25 °C, ppm): 8.52 (s, 1H, CH=N^4^); 8.33 (s, 2H, NH_2_); 8.30 (s, 1H, CH=N^1^); 7.93 (dd, 1H, *J* = 1.34, *J* = 8.72, *x*); 7.74 (dd, 1H, *J* = 1.68, *J* = 8.05, *d*); 7.55 (dd, 1H, *J* = 1.68, *J* = 8.05, *s*); 7.45 (td, 1H, *J* = 1.68, *J* = 6.71, *J* = 7.04, *b*); 7.37 (ddd, 1H, *J* = 1.34, *J* = 1.68, *J* = 6.71, *z*); 7.33 (td, 1H, *J* = 1.68, *J* = 6.71, *J* = 7.05, *q*); 7.00 (dd, 1H, *J* = 1.34, *J* = 8.72, *w*); 6.98 (d, 1H, *J* = 8.73, *p*); 6.91 (d, 1H, *J* = 8.73, *a*); 6.71 (t, 1H, *J* = 7.04, *J* = 7.72, *c*); 6.63 (t, 1H, *J* = 7.04, *J* = 7.38, *r*); 6.59 (ddd, 1H, *J* = 1.01, *J* = 1.68, *J* = 6.71, *y*); 2.71 (s, 3H, S-CH_3_).

##### 2.2.3.3 Characterization Data of **Complex II**

Color: red; yield: 49%; mp (°C): 179–180; anal. calcd for C_22_H_18_N_6_NiO_6_S (553.17 g/mol): calculated: C, 47.77; H, 3.28; N, 15.19; S, 5.81%. Found: C, 47.12; H, 3.54; N, 15.08; S, 5.29%. IR (cm^–1^): ν­(C=N) 1625, ν­(C–O) 1606, 1584; ν­(–NH) 1160, 1123; 3449, 3332; ν­(-NO_2_)­1540, 1388. UV–vis (5 × 10^–5^ M, CHCl_3_) (λ (ε)): 227 (11000), 242.5 (18240), 302.5 (9180), 326 (8720), 393 (7600), 475.5 (2700), 549 (1560). ^1^H NMR (DMSO-*d*_6_, 25^◦^C, ppm): 8.80–8.46 (broad, s, 2H, NH_2_); 8.77 (d, 1H, *J* = 2.93, *w*); 8.50 (s, 1H, CH=N^4^); 8.29 (s, 1H, CH=N^1^); 8.14 (dd, *J* = 2.93, *J* = 9.76, 1H, *y*); 7.74 (dd, *J* = 1.46, *J* = 8.29, 1H, *d*); 7.53 (dd, *J* = 1.95, *J* = 8.59, 1H, *s*); 7.44 (ddd, *J* = 1.47, *J* = 6.35, *J* = 8.3, 1H, *b*); 7.29 (ddd, *J* = 1.96, *J* = 6.84, *J* = 8.79, 1H, *q*); 7.09 (d, 9.76, 1H, *z*); 6.97 (d, *J* = 8.29, 1H, *p*); 6.89 (d, *J* = 8.29, 1H, *a*); 6.70 (ddd, *J* = 1.46, *J* = 7.32, *J* = 8.3, 1H, *c*); 6.64 (t, *J* = 6.83, 1H, *r*); 2.70 (s, 3H, S-CH_3_).

### 2.3 X-ray Analysis

Intensity data of **Complex I** and **Complex II** were collected with a STOE IPDS II diffractometer at room temperature by using graphite-monochromated Mo Kα radiation by applying the ω-scan method. Data collection and cell refinement were carried out using X-AREA,^39^ while data reduction was applied using X-RED32.^41^ The structures were solved by direct methods with SIR2019 ^40^ and refined by means of the full-matrix least-squares calculations on *F* ^2^ using SHELXL-2018.^41^ All H atoms were located in a difference electron-density map and then treated as riding atoms in geometrically idealized positions, with N–H = 0.86 (NH), C–H = 0.93 (CH), and 0.96 Å (CH_3_) and with *U*_iso_(H) = *kU*_eq_(C), where *k* = 1.5 for the methyl atom and 1.2 for all other H atoms. In both compounds, atoms S1/N2/C8 were disordered over the axis passing through the Ni1 and C9 atoms. The refined site-occupancy factors of the disordered parts are 0.788(5)/0.212(5)% for **Complex I** and 0.610(3)/0.390(3)% for **Complex II**. In the following, geometric parameters for the minor parts of the disordered fragments are listed in square brackets. Crystal data, data collection, and structure refinement details are given in Table 1. Molecular graphics were generated by using OLEX2.^42^

**Table 1:** Crystal Data and Structure Refinement Parameters for **Complex I** and **Complex II**

parameters	**Complex I**	**Complex II**
CCDC depository	2014130	1977073
color/shape	red/plate	dark red/prism
chemical formula	[Ni(C_16_H_13_N_3_O_2_S)]·(C_6_H_6_N_2_O_2_)	[Ni(C_16_H_13_N_3_O_2_S)]·(C_6_H_5_N_3_O_4_)
formula weight	508.19	553.19
temperature (K)	296(2)	296(2)
wavelength (Å)	0.71073 Mo Kα	0.71073 Mo Kα
crystal system	monoclinic	monoclinic
space group	*P*2_1_/*c* (no. 14)	*P*2_1_/*c* (no. 14)
unit cell parameters		
*a*, *b*, *c* (Å)	11.9960(6), 22.853(2), 8.0381(10)	13.5307(7), 13.7903(10), 13.4000(6)
α, β, γ (deg)	90, 91.000(7), 90	90, 114.014(4), 90
volume (Å^3^)	2203.2(4)	2283.9(2)
*Z*	4	4
*D*_calcd_ (g/cm^3^)	1.532	1.609
μ (mm^–1^)	1.016	0.995
absorption correction	integration	integration
*T*_min._, *T*_max._	0.7583, 0.9497	0.7308, 0.8638
*F*_000_	1048	1136
crystal size (mm^3^)	0.51 × 0.16 × 0.04	0.41 × 0.32 × 0.17
diffractometer	STOE IPDS II	STOE IPDS II
measurement method	ω scan	ω scan
index ranges	–14 ≤ *h* ≤ 13, −27 ≤ *k* ≤ 27, −9 ≤ *l* ≤ 9	–17 ≤ *h* ≤ 17, −18 ≤ *k* ≤ 18, −16 ≤ *l* ≤ 17
θ range for data collection (deg)	1.782 ≤ θ ≤ 25.048	2.213 ≤ θ ≤ 27.915
reflections collected	11,703	27,048
independent/observed reflections	3881/1735	5432/3237
*R*_int._	0.1540	0.1057
refinement method	full-matrix least-squares on *F* ^2^	full-matrix least-squares on *F* ^2^
data/restraints/parameters	3881/80/326	5432/80/353
goodness-of-fit on *F* ^2^	1.017	1.196
final *R* indices [*I* > 2σ(*I*)]	*R*_1_ = 0.0926, w *R*_2_ = 0.1702	*R*_1_ = 0.0856, w *R*_2_ = 0.1434
*R* indices (all data)	*R*_1_ = 0.1985, w *R*_2_ = 0.2118	*R*_1_ = 0.1442, w *R*_2_ = 0.1623
Δρ_max._, Δρ_min._ (e/Å^3^)	0.41, −0.39	0.27, −0.21

### 2.4 Computational Procedure

Quantum chemical computations for the starting thiosemicarbazone compound and **Complex I** and **Complex II** were carried out with the GaussView 5 ^43^ molecular visualization program and the Gaussian 09 program package.^44^ The structural, spectroscopic, and electronic properties were obtained using the HSEH1PBE density functional method^45–48^ with the cc-pVDZ basis set^49^ for C, H, N, O, and S atoms and the LanL2DZ basis set^50–52^ for the Ni atom. The calculated vibrational wavenumbers without imaginary frequencies were scaled by 0.962. The ^1^H chemical shifts were obtained via the gauge-independent atomic orbital (GIAO) approach,^53,54^ while the electronic absorption spectra were computed using the time-dependent density functional theory (TD-DFT)^55,56^ at the same level. In these calculations, solvent effects were considered by using the conductor-like polarizable continuum model (CPCM).^57–59^

### 2.5 Electrochemical Studies

Cyclic voltammetry (CV) was used for electrochemical characterizations. A Gamry Reference 600 potentiostat/galvanostat utilizing a three-electrode configuration at 25 °C was used for the electrochemical measurements. A glassy carbon electrode (GCE), a Pt wire, and a Ag/AgCl electrode served as the working, counter, and reference electrodes, respectively. Dimethyl sulfoxide (DMSO) containing 0.10 mol·dm^–3^ tetrabutylammonium perchlorate (TBAP) was used as the electrolyte. In situ spectroelectrochemical measurements were carried out by utilizing a three-electrode configuration of a thin-layer quartz spectroelectrochemical cell by using an OceanOptics QE65000 diode array spectrophotometer. The working electrode was a semitransparent Pt tulle.

## 3 Results and Discussion

### 3.1 Synthesis

Thiosemicarbazones containing more than one donor atom (N, S, and additional donor atoms) have attracted significant attention in coordination chemistry. When salicylaldehyde-*S*-methyl-isothiosemicarbazone reacts with nickel­(II) chloride and if the thiosemicarbazone compound—whether N-substituted or S-substituted—subsequently reacts with a second ligand molecule containing an N donor atom, the latter ligand generally coordinates to the metal center as a coligand via the N donor atoms. This coordination pattern is observed in compounds with coligands such as ammonia,^60–65^ tmen,^66,67^ pyridine,^68–72^ 4-methylpyridine,^73,74^ 4-aminopyridine,^74^ 2,2′-bipyridine,^75–80^ imidazole,^71,73,81–83^ benzimidazole,^84^ and 1,10-phenanthroline.^85–87^ Based on this principle, when a one-pot reaction was performed by mixing an amine compound (2,4-dinitroaniline), salicylaldehyde-*S*-methylthiosemicarbazone, and nickel­(II) chloride, it was anticipated that the amine group would bind to the nickel center as a coligand. Another possibility is that the thiosemicarbazone may form a triazole heterocycle, or in another scenario, it may fragment as described in our previous work. In this study, while *N*(1)-salicylidene-*S*-methyl-isothiosemicarbazone fragmented into salicylaldehyde in the presence of nitro-substituted aniline in one pot, the fragmented salicylaldehyde subsequently bonded to the free amino group of the thiosemicarbazone ligand, acting as a tetradentate chelating agent to the metal atom, thus simultaneously forming the template complex. However, the nitro-substituted aniline ligand preferred to form second-sphere coordination interactions rather than directly coordinating to nickel, resulting in the formation of a nickelophilic complex. The Cambridge Structural Database^88^ contains 11 examples of Nickelophines, including ONNO coordination mode, so far. However, only one nickel-based thiosemicarbazone compound has been reported, which is bis­(3-((((methylsulfanyl)­(((2-oxidophenyl)­methylidene)­amino) methylidene)­hydrazinylidene) methyl)-2*H*-1-benzopyran-4-olato)-dinickel­(II) (Table S1). To the best of our knowledge, there are no results in the literature regarding the synthesis of a nickelophilic thiosemicarbazone with semiconductor and solvatochromic properties, up until now.

The product yields were 47% for **Complex I** and 49% for **Complex II**. Both complexes were obtained in red crystal form, and they were very soluble in diethyl ether, dichloromethane, DMF, and DMSO, while their solubility in ethanol and methanol was lower. The complexes were not soluble in water. The structures of the complexes were characterized by elemental analysis, FT-IR, UV–vis, and ^1^H NMR spectroscopy. Magnetic susceptibility measurements were performed, and the results clearly indicate that the compounds are diamagnetic in nature. This suggests that there is no significant magnetic coupling between the Ni centers, and any metallophilic Ni···Ni interactions present do not lead to a paramagnetic behavior. Additionally, their structures were elucidated by using the X-ray diffraction method.

### 3.2 Structural Characterization (Experimental vs Theoretical Structures)

The solid-state structures of **Complex I** and **Complex II** have been unambiguously determined by single-crystal X-ray analysis. Molecular structures of **Complex I** and **Complex II** are presented in Figures 1a and 2a, respectively, while selected experimental and theoretical geometric parameters are quoted in Table 2.

**Figure 1:**
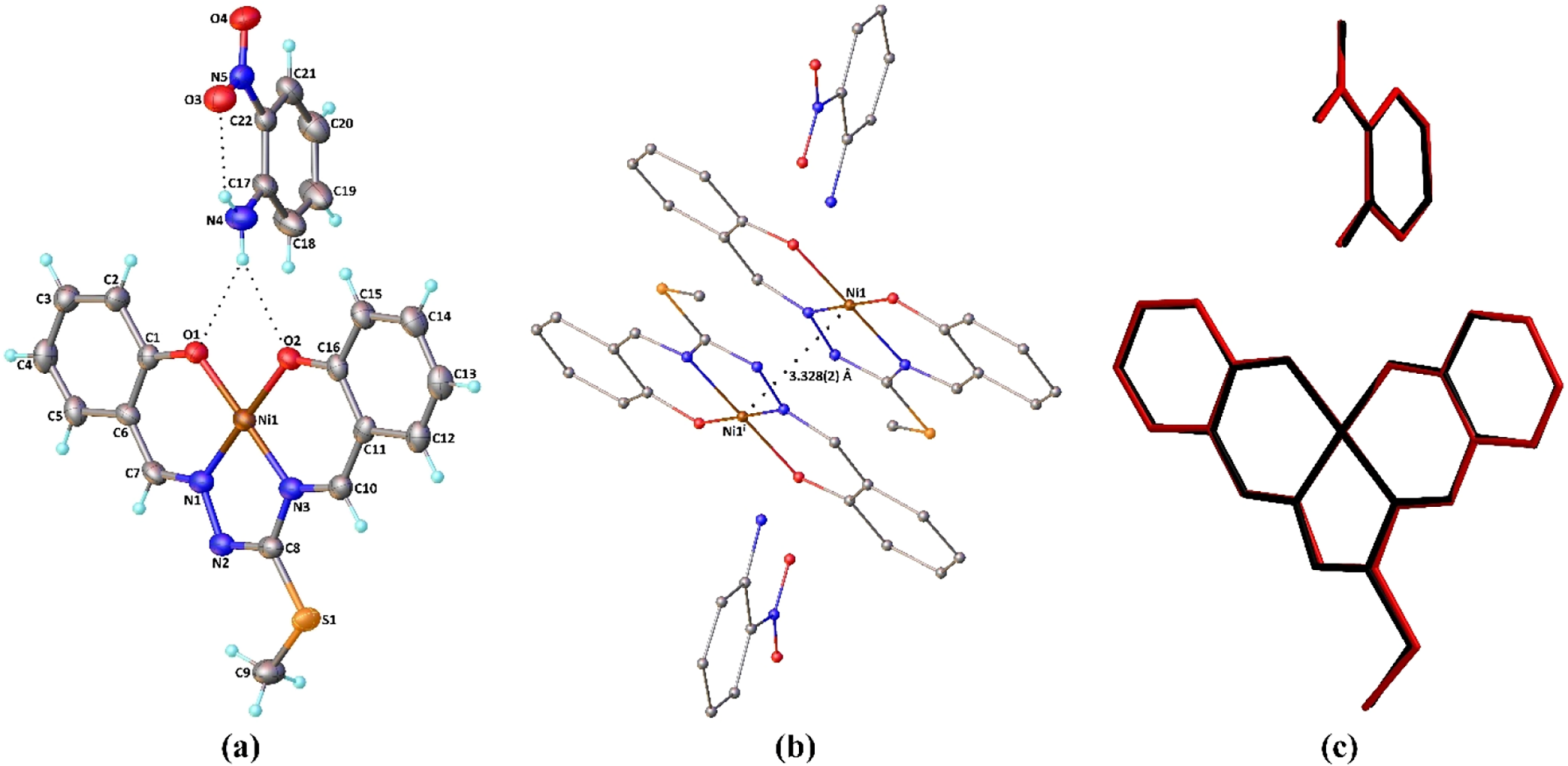
(a) Molecular structure of **Complex I** showing the atom-labeling scheme. H atoms are shown as small spheres of arbitrary radii; only a major part of the disordered fragment is shown for clarity. (b) Ni···Ni interaction generated by inversion (symmetry code: ^i^1 – *x*, 1 – *y*, 2 – *z*). Hydrogen atoms are omitted for clarity. (c) Atom-by-atom superimposition of the structures calculated (red) over the X-ray structure (black). Hydrogen atoms are omitted.

**Figure 2:**
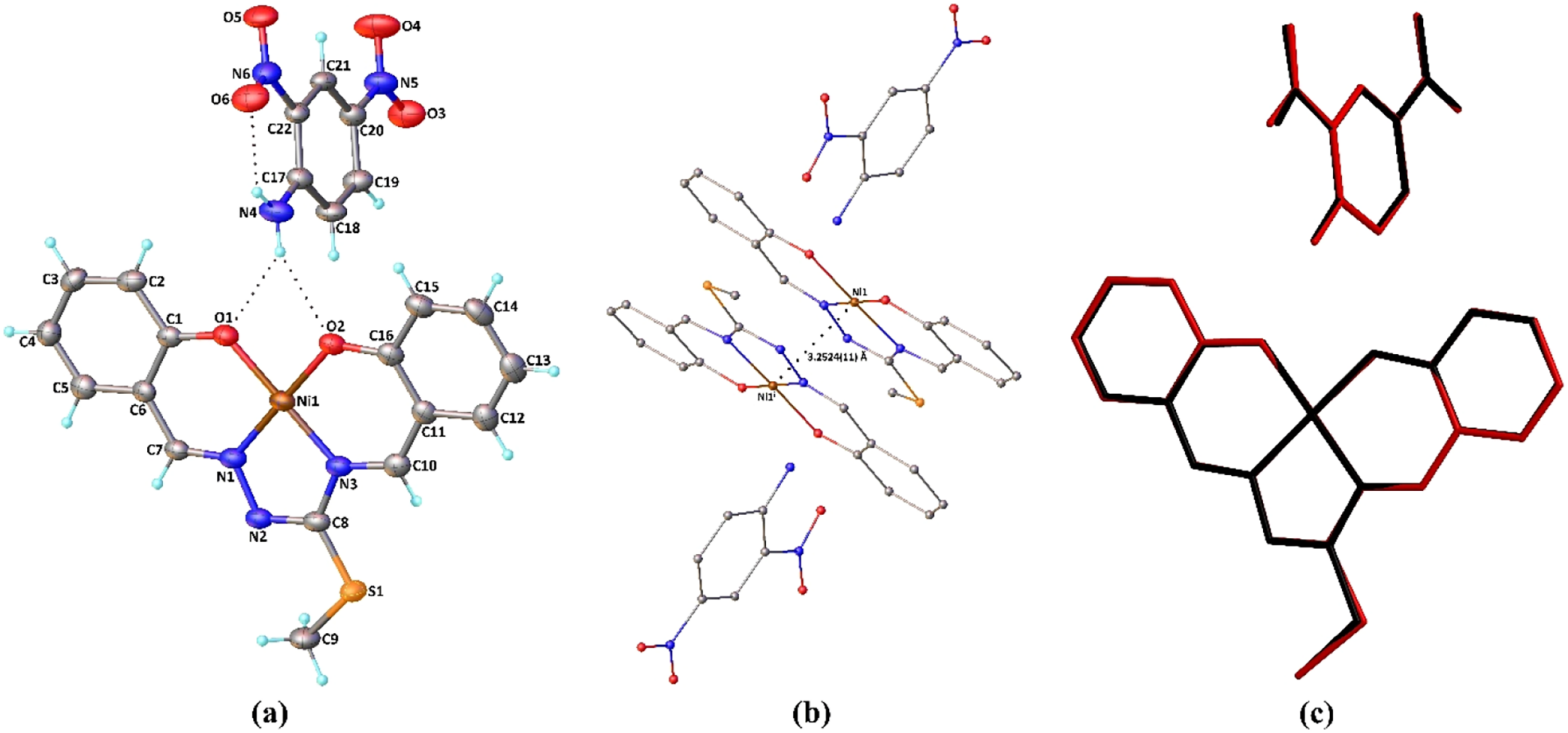
(a) Molecular structure of **Complex II** showing the atom-labeling scheme. H atoms are shown as small spheres of arbitrary radii; only a major part of the disordered fragment is shown for clarity. (b) Ni···Ni interaction generated by inversion (symmetry code: ^i^1 – *x*, 1 – *y*, 2 – *z*). Hydrogen atoms are omitted for clarity. (c) Atom-by-atom superimposition of the structures calculated (red) over the X-ray structure (black). Hydrogen atoms are omitted.

**Table 2:** Selected Geometric Parameters for **Complex I** and **Complex II**

	**Complex I**		**Complex II**	
parameters	X-ray	DFT	X-ray	DFT
bond lengths (Å)				
Ni1–O1	1.830(6)	1.847	1.835(4)	1.849
Ni1–O2	1.851(6)	1.841	1.838(4)	1.845
Ni1–N1	1.833(7)	1.846	1.817(4)	1.838
Ni1–N3	1.818(8)	1.859	1.821(4)	1.852
S1–C8	1.722(12) [1.721(12)]	1.759	1.755(9) [1.755(9)]	1.758
S1–C9	1.760(8) [1.759(8)]	1.809	1.854(4) [1.856(4)]	1.809
O1–C1	1.315(10)	1.301	1.310(6)	1.305
O2–C16	1.304(10)	1.285	1.332(7)	1.289
N1–C7	1.268(9)	1.306	1.299(5)	1.306
N1–N2	1.297(10) [1.298(10)]	1.371	1.356(8) [1.356(8)]	1.372
N2–C8	1.265(12) [1.264(12)]	1.295	1.274(8) [1.274(8)]	1.294
N3–C8	1.522(12) [1.524(12)]	1.391	1.461(10) [1.462(10)]	1.393
N3–C10	1.321(10)	1.319	1.315(5)	1.318
O3–N5	1.221(11)	1.235	1.204(6)	1.220
O4–N5	1.222(10)	1.218	1.198(6)	1.218
O5–N6			1.215(6)	1.215
O6–N6			1.220(6)	1.230
N4–C17	1.347(13)	1.347	1.325(6)	1.337
N5–C22	1.428(13)	1.442		
N5–C20			1.453(7)	1.453
N6–C22			1.462(7)	1.449
bond angles (deg)				
N1–Ni1–N3	83.4(4)	83.14	83.3(2)	83.24
N1–Ni1–O1	96.5(3)	94.99	96.20(17)	95.17
N3–Ni1–O1	179.1(3)	178.04	178.08(16)	178.40
N1–Ni1–O2	176.8(3)	177.87	178.58(18)	178.29
N3–Ni1–O2	93.4(3)	94.81	95.39(17)	95.09
O1–Ni1–O2	86.7(3)	87.07	85.16(18)	86.50
C8–S1–C9	101.5(5) [101.6(5)]	100.19	100.3(4) [100.2(4)]	100.17
C7–N1–N2	112.0(8) [108.3(8)]	116.53	111.1(5) [110.0(5)]	116.18
N1–N2–C8	108.1(11) [108.1(11)]	110.77	104.2(8) [104.2(8)]	110.65
N2–C8–N3	119.9(11) [119.8(11)]	119.12	123.6(9) [123.6(9)]	118.86
N2–C8–S1	122.6(10) [122.6(10)]	120.66	119.1(9) [119.0(9)]	120.83
N3–C8–S1	117.5(8) [117.6(8)]	120.22	117.3(6) [117.3(6)]	120.31
C8–N3–C10	124.0(8) [127.9(7)]	123.01	124.8(5) [125.9(5)]	122.73
O3–N5–O4	119.9(10)	122.53	122.7(5)	124.90
O5–N6–O6			123.3(5)	123.34
O3–N5–C22	119.6(11)	118.71		
O4–N5–C22	120.5(11)	118.75		
O3–N5–C20			119.3(5)	117.25
O4–N5–C20			117.9(6)	117.85
O5–N6–C22			119.0(5)	118.46
O6–N6–C22			117.6(5)	118.20

In the asymmetric unit of the cocrystal compounds, there is a mononuclear nickel complex and a 2-nitroaniline molecule in **Complex I** and a 2,4-dinitroaniline molecule in **Complex II**. The complex part of the compounds is the same and composed of an *S*-methyl-*N* ^1^,*N* ^4^-bis­(salicylidene)-isothiosemicarbazide ligand, whose structure was reported by Purwell et al. in 1985,^89^ and a Ni­(II) metal center. As shown in Figures 1a and 2a, the central Ni atom is tetracoordinated in a square-planar geometry. The two phenolato oxygen atoms and two azomethine nitrogen atoms of the doubly deprotonated *ONNO* tetradentate Schiff-base ligand occupy the corners of a square plane. The four-coordinate geometry index (τ_4_)^90^ is found to be 0.03 for **Complex I** and 0.02 for **Complex II** both experimentally and theoretically and indicates a slightly distorted square-planar geometry around the metal atom. The bond distances between the metal and donor atoms span a narrow range from 1.818(8) to 1.851(6) Å for **Complex I** and from 1.817(4) to 1.838(4) Å for **Complex II**. The metal–ligand bond distances are calculated in the ranges of 1.841–1.859 Å for **Complex I** and 1.838–1.852 Å for **Complex II**. However, the coordination bond distances are typical of related square-planar Ni complexes.^91–94^ The *trans* angles, varying from 176.8(3) to 179.1(3)°, and the *cis* angles, changing from 83.3(2) to 96.5(3)°, confirm the distortion of the coordination around the nickel ions. The alteration in the *cis* and *trans* angle values ranges from 83.14 to 95.17° and from 177.87 to 178.40° in the theoretical structures, respectively. When the bond lengths in the free and coordinated isothiosemicarbazide ligands are compared, it is seen that C–O bonds shorten upon the formation of Ni–O bonds, while there are slight changes of 0.01–0.03 Å for the remaining bonds.

Inspection of Figures 1b and 2b displays a similarity between the X-ray and the DFT geometries by superimposing them. Harmony between the calculated and experimentally determined X-ray structures is excellent, with root-mean-square deviation (RMSD) values of 0.083 and 0.052 Å in **Complex I** and 0.082 and 0.061 Å in **Complex II** for the complex and organic parts of the cocrystals, respectively.

The supramolecular features of the compounds are also similar. In their molecular structures, intramolecular N–H···O contact within the organic molecule leads to the formation of a six-membered ring (Figures 1a and 2a) with graph-set descriptor *S*(6).^95^ Besides, the organic moiety is connected to the complex fragment by two intermolecular N–H···O hydrogen bonds (Table 3), forming an *R*_1_^2^(4) ring. In their crystal structures, the cocrystal compounds stack along the *c* axis and forms centrosymmetric pairs in which the complex parts at (*x*, *y*, *z*) and (1 – *x*, 1 – *y*, 2 – *z*) are linked to each other via π···π stacking interactions between the six-membered chelate rings with a centroid–centroid distance of 3.377(4) Å in **Complex I** and 3.310(3) Å in **Complex II**. In this arrangement, the Ni···Ni interaction distance is 3.328(2) Å in **Complex I** and 3.2524(11) Å in **Complex II**. Metallophilic contacts are limited to isolated interactions between two inversion-related complexes with no extended Ni···Ni chains. In our study, we have reviewed 11 nickelophilic complexes exhibiting Ni···Ni interactions in the Cambridge Structural Database (CSD)^88^ within the ONNO coordination mode. The Ni···Ni interaction distances (in Å) in these complexes range from 3.251 to 3.419 Å, as detailed in Table S1 in the Supporting Information and the zigzag motif (Figures 3 and 4). In **Complex I**, this connection is achieved by interaction between the benzene and five-membered chelate rings with a centroid–centroid distance of 3.567(5) Å [3.624(6) Å] and by interaction between the six-membered chelate rings with a centroid–centroid distance of 3.731(4) Å in the molecule at (*x*, *y*, *z*) and (1 – *x*, 1 – *y*, 1 – *z*). In the case of **Complex II**, interaction between the benzene and six-membered chelate rings in the molecule at (*x*, *y*, *z*) and (1 – *x*, −1/2 + *y*, 3/2 – *z*) with a centroid–centroid distance of 3.635(3) Å is responsible for this connection.

**Figure 3:**
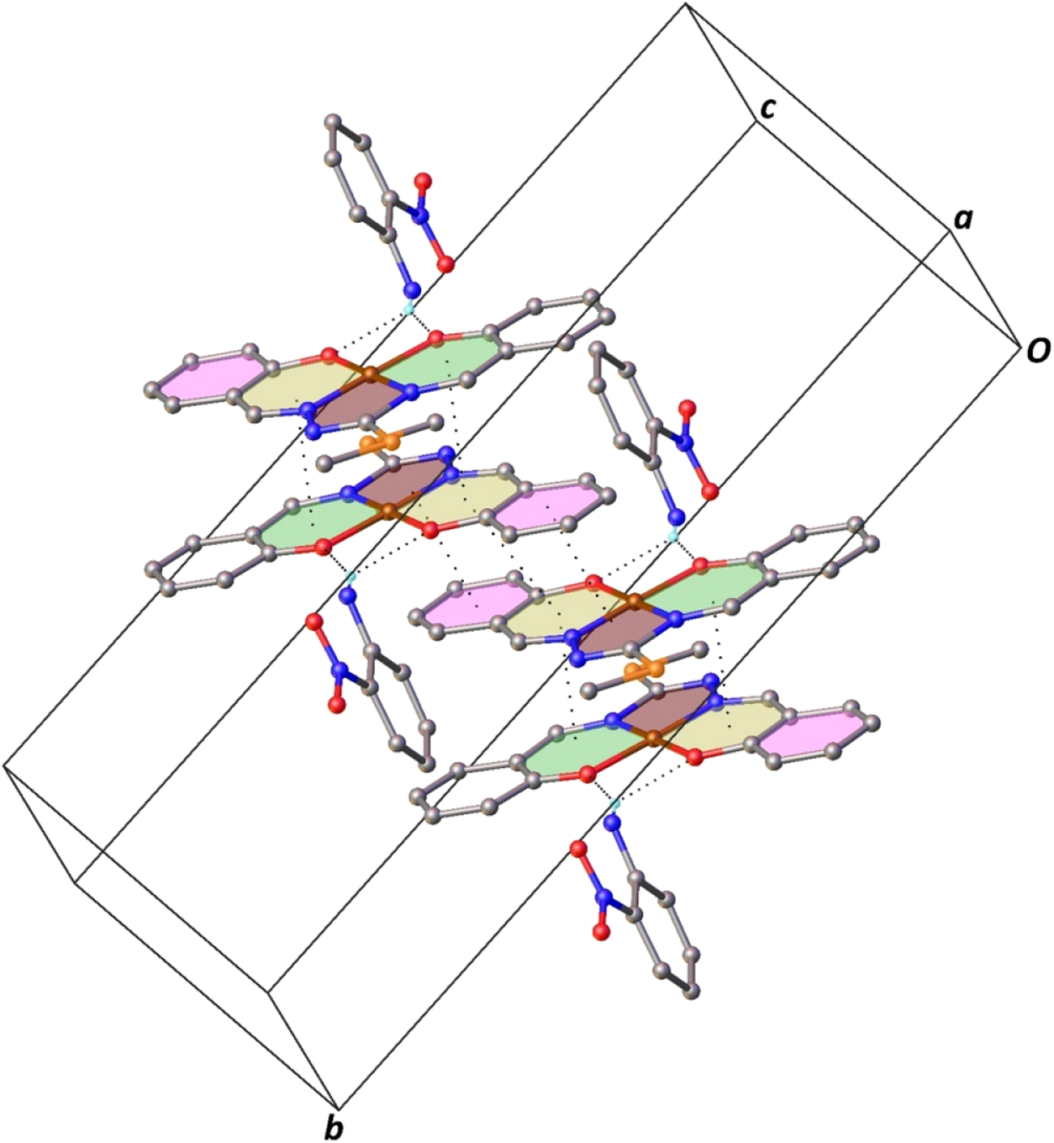
Part of the crystal structure of **Complex I** showing the intermolecular N–H···O and π···π stacking interactions. For the sake of clarity, only H atoms involved in hydrogen bonding have been included.

**Figure 4:**
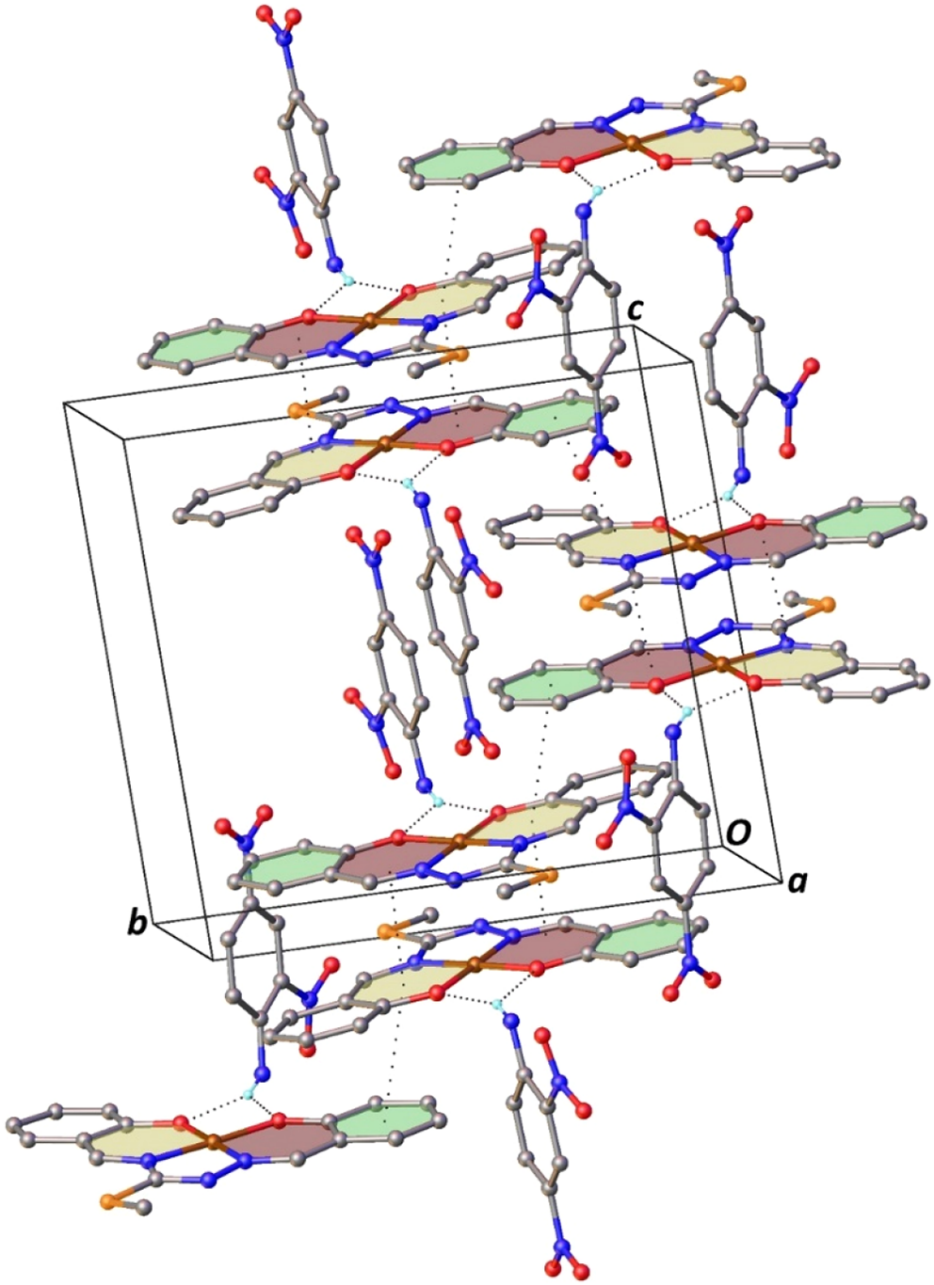
Part of the crystal structure of **Complex II** showing the intermolecular N–H···O and π···π stacking interactions. For the sake of clarity, only H atoms involved in hydrogen bonding have been included.

**Table 3:** Hydrogen Bonding Geometry for **Complex I** and **Complex II**

D–H···A	D–H (Å)	H···A (Å)	D···A (Å)	D–H···A (deg)
**Complex I**				
N4–H4A···O3	0.86	2.04	2.636(12)	126
N4–H4B···O2	0.86	2.30	3.102(11)	156
N4–H4B···O1	0.86	2.48	3.177(10)	139
**Complex II**				
N4–H4A···O6	0.86	2.01	2.613(6)	127
N4–H4B···O2	0.86	2.25	3.050(6)	155
N4–H4B···O1	0.86	2.40	3.054(6)	133

Finally, each centrosymmetric pair interacts with an adjacent one through weaker π···π interactions, creating a zig-zag motif.

#### 3.2.1 FT-IR Spectroscopy

The IR spectrum of the starting material showed bands at 3458, 3278, and 1635 cm^–1^, which were attributed to ν_asym_(NH), ν_sym_(NH), and δ­(NH_2_) vibrations, respectively. These bands were expected to disappear in the IR spectra of the complex due to the chelation and were calculated at 3534, 3421, and 1635 cm^–1^, respectively. However, the bands observed at 3479, 3302 cm^–1^ for **Complex** **I** and at 3449, 3332 cm^–1^ for **Complex II** were attributed to ν_asym_(NH) and ν_sym_ (NH) vibrations of 2-nitroaniline and 2,4-dinitroaniline, respectively. In the theoretical spectrum, these bands were predicted at 3511 and 3326 cm^–1^ for **Complex I** and at 3502 and 3296 cm^–1^ for **Complex II** (Figures S1–S3in the Supporting Information).

The imine band of the starting material was observed at 1617 and 1602 cm^–1^, while these vibrations were theoretically observed at 1644 and 1599 cm^–1^, respectively. After chelating, a new sharp intensity band was observed in the range of 1625–1584 cm^–1^ belonging to a new imine group, (N^4^=C), which resulted from the condensation of the thioamide nitrogen (N^4^) with a second aldehyde. The monitored azomethine stretching vibration bands at 1604, 1599, and 1578 cm^–1^ in the spectrum of **Complex I** and at 1625, 1606, 1584 cm^–1^ in the spectrum of **Complex** **II** appeared at 1612, 1596, and 1557 cm^–1^ for **Complex I** and at 1611, 1596, and 1558 cm^–1^ for **Complex II** in the theoretical spectrum, respectively.^94,98^

The peaks calculated at 1342 and 1312 cm^–1^ for **Complex I** and at 1337 and 1308 cm^–1^ for **Complex II** were assigned to C–O vibrations that have been observed at 1140 and 1121 cm^–1^ for **Complex I** and at 1160 and 1123 cm^–1^ for **Complex II** in the FT-IR spectra, respectively. The absorptions at 1328 and 1256 cm^–1^ (C–N stretching of –NH_2_) and at 1540 and 1388 cm^–1^ (asymmetric and symmetric stretching of –NO_2_, respectively) indicate the presence of a nitroaniline group.^96^ These bands were recorded at 1480, 1395, 1600, and 1353 cm^–1^ for **Complex I** and at 1484, 1404, 1612, and 1379 cm^–1^ for **Complex II**, in the theoretical spectrum, respectively.

#### 3.2.2 UV–Vis Spectroscopy

The UV–vis spectra of the starting material and complexes were recorded from 200 to 800 nm. The UV–vis spectra of the starting material in CHCl_3_ showed five bands at 239.5, 293, 304.5, 333.5, and 346.5_sh_ nm, which could be assigned to the π–π* and n–σ* transitions of the aromatic ring, phenol, amine, thioether, and n–π* transition of the azomethine group on the thiosemicarbazone, respectively.

In the UV–vis spectra of **Complex I**, π–π* transitions of intramolecular charge transfer were observed at 240.5, 281, and 300 nm, and n–π* transitions, which belong to the azomethine group, were recorded at 322.5 nm. Charge transfer transitions, which are from ligand-to-metal and d–d transitions were recorded at 395 and 549.5 nm, respectively. The UV–vis spectrum of **Complex II** was similar to that of **Complex I**. The bands were observed at 227, 242.5, 302.5, 326, 393, 475.5, and 549.5 nm. The bands at 228, 280, and 474 nm were assigned to the nitro-substituted aniline in the crystal structure.^97^ When the starting material was connected to salicylaldehyde, a conjugated chelate structure with a π-electron system was formed. As a result, both increased conjugation and coordination to the metal atom through the azomethine groups of the chelate caused absorptions to shift to longer wavelengths (Figures S4–S6 in the Supporting Information)..

#### 3.2.3 ^1^H NMR Spectroscopy

In the ^1^H NMR spectrum of the starting material, the proton signals of the hydroxyl groups corresponding to the *cis*–*trans* isomers were observed at 11.58 and 10.69 ppm. After chelation, these signals disappeared from the spectra of the complexes. Additionally, the protons of the imine groups appeared at the expected chemical shift values for both complexes. In the spectra of the complexes, the proton signals of the aromatic groups were observed in the range of 8.14–6.59 ppm.^98^ The NH_2_ signal corresponding to the aniline group in **Complex I** was detected at 8.33 ppm. Similarly, the amine group signal in **Complex II** was broadly observed in the range of 8.80–8.46 ppm. During the spectral analysis of the complexes, a mismatch was observed between the integral values and the expected number of protons in the molecular structure. This discrepancy was explained through X-ray analysis, which confirmed the structures of the complexes (Figures S7–S9 in the Supporting Information).

#### 3.2.4 TD-DFT Method

After the absorption spectra of the compounds were calculated by the TD-DFT method, the major contributions from molecular orbitals (HOMO: H, LUMO: L) to the electronic transitions are designated with the aid of the GaussSum program.^99^ All of the related molecular orbitals are shown in Figure 5. TD-DFT calculations predict absorptions at 234 nm [major contributions: H→L+1 (64%), H–3→L (13%), and H–4→L (10%)], 247 nm [major contributions: H-3→L (83%) and H→L+1 (14%)], 289 nm [major contribution: H–1→L (91%)], and 331 nm [major contribution: H→L (97%)] for the starting thiosemicarbazone compound and at 485 nm [major contribution: H→L (73%) and H–1→L (24%)] for **Complex I** and at 486 nm [major contribution: H→L (97%)] for **Complex II**. In addition, the value of the energy separation between the H and L is found to be 3.75 eV for the starting material, 2.32 eV for **Complex I**, and 2.62 eV for **Complex II**.

**Figure 5:**
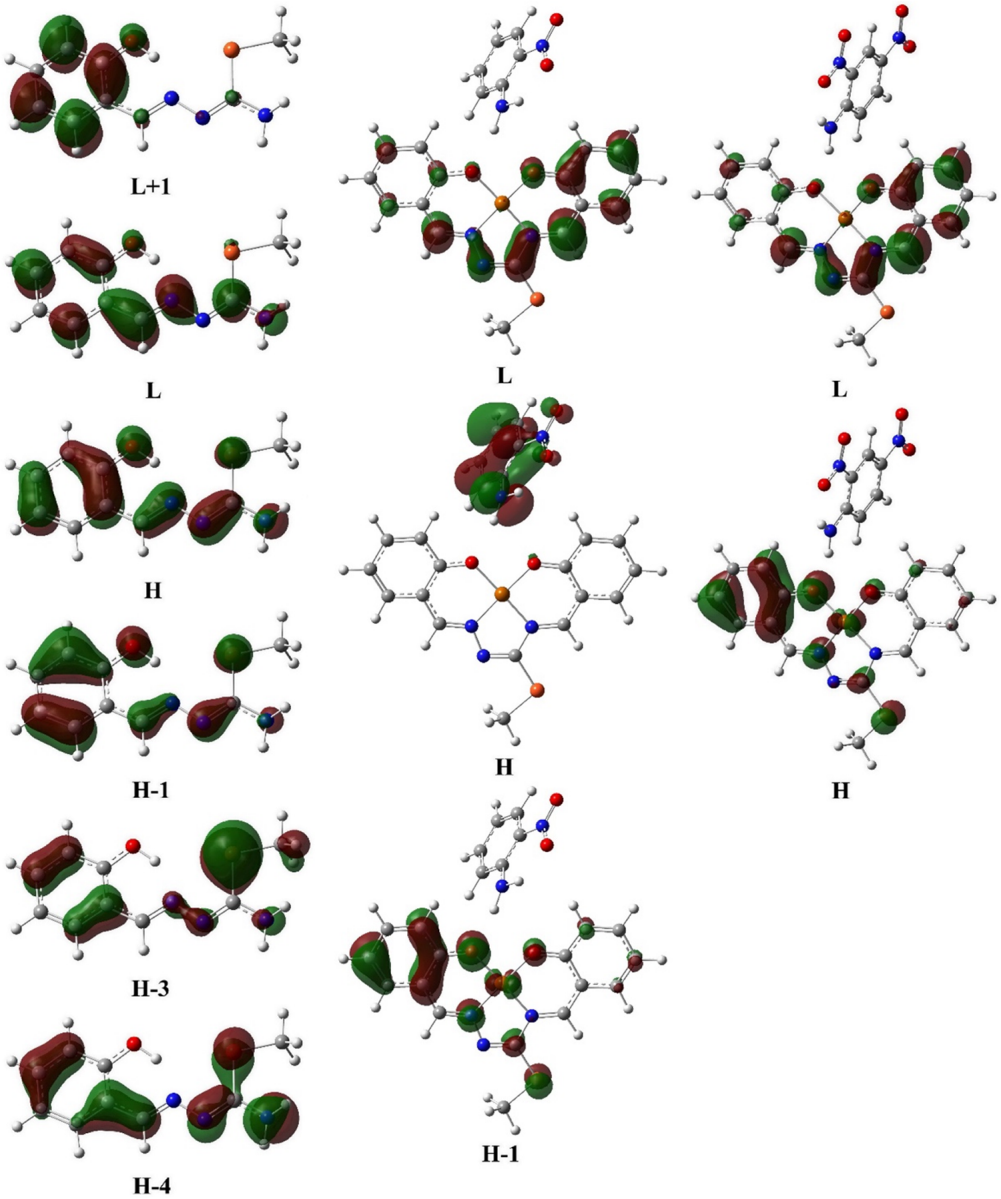
Selected molecular orbital surfaces of the starting material, **Complex I**, and **Complex II** (H: HOMO, L: LUMO).

### 3.3 Nonlinear Optical Properties

#### 3.3.1 Theoretical Optical Properties

The calculations of the mean linear polarizability (α) and the mean first hyperpolarizability (β) from the Gaussian output have been explained in detail previously.^100^ The calculated values of α and β are 59.92 Å^3^ and 52.33 × 10^–31^ cm^5^/esu for **Complex I** and 60.02 Å^3^ and 104.12 × 10^–31^ cm^5^/esu for **Complex II**, respectively. Urea and p-nitroaniline are prototypical molecules used in the study of the NLO properties of molecular systems. Therefore, they are used as reference molecules in NLO studies. However, since **Complex I** contains a 2-nitroaniline and **Complex II** contains a 2,4-nitroaniline, α and β values of these molecules were chosen for comparison. The calculated values of α and β for urea, 2-nitroaniline, and 2,4-nitroaniline at the same level are 3.88 Å^3^ and 7.71 × 10^–31^ cm^5^/esu, 12.51 Å^3^ and 22.91 × 10^–31^ cm^5^/esu, and 15.17 Å^3^ and 71.47 × 10^–31^ cm^5^/esu, respectively. It is seen that the α and β values of both complexes are much higher than those of urea. The obtained α and β values for **Complex I** are 4.8 and 2.3 times higher than those for 2-nitroaniline, while the obtained α and β values for **Complex II** are 4.0 and 1.5 times higher than those for 2,4-nitroaniline. According to these values, both complexes can be further used as good candidates for NLO materials.

#### 3.3.2 Experimental Optical properties

The band gaps of the synthesized complexes were determined using diffuse reflectance spectra, requiring testing the possibility of being an optoelectronic device (Figure 6). Tauc’s relation was applied to calculate the band gap values, as given by eq 1

1 $${\left(\alpha h\nu \right)}^{n}=A\left(h\nu -E_{g}\right)$$

where *A* is the band edge parameter, and the value of *n* determines the nature of the optical transition (*n* = 1/2 indicates a direct allowed transition and *n* = 2 indicates an indirect allowed transition). *E*_g_ is the optical band gap, and *h* is Planck’s constant. The direct energy gap was determined by plotting a graph of (α*h*ν)^2^ versus *h*ν and extrapolating the straight-line portion to zero.^101^

**Figure 6:**
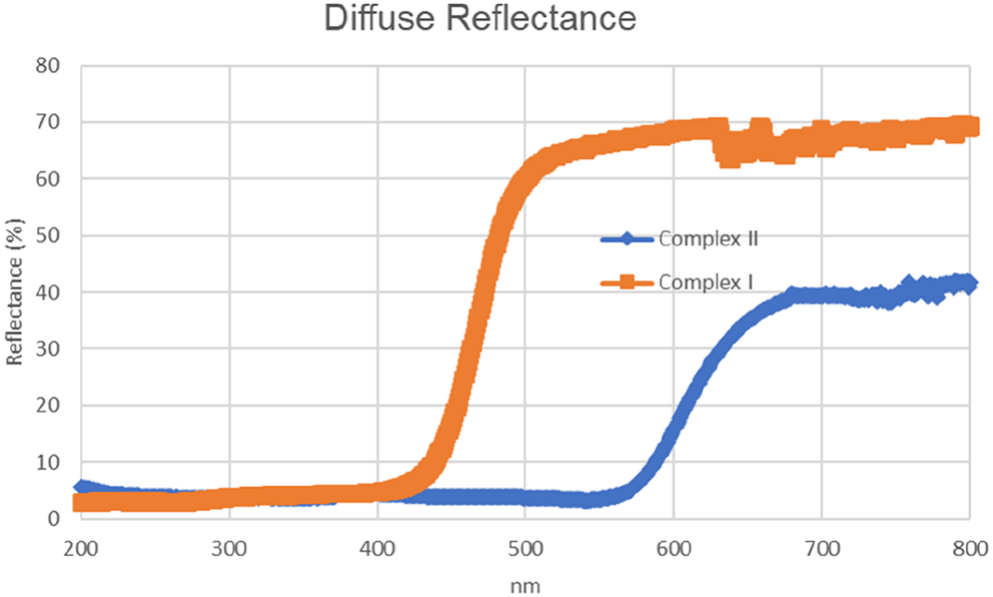
Diffuse reflectance spectrum of **Complex I** and **Complex II**.

The optical diffuse reflectance spectra of **Complex I** and **Complex II** showed reflectance on the long wavelengths and exhibited 1.962 and 1.818 eV absorption edges, respectively, in the UV–vis spectrum. Since these values fall within the typical band gap range of semiconductors, we can conclude that the complexes exhibit promising potential for various semiconductor applications, such as light-emitting diodes (LEDs), solar cells, and transistors.^102,103^

The highest occupied molecular orbital–lowest unoccupied molecular orbital (HOMO–LUMO) energy gap (band gap) is a key physical property of a molecule that can create the potential for interacting electrons in energy levels. A smaller band gap value implies an easier excitation of electrons, which increases the molecule’s sensitivity to light (photosensitivity).

### 3.4 Solvent Effects on the UV–Vis Absorption Spectra

The UV–vis spectra of the complexes were recorded in the range of 200–800 nm. The UV spectra of **Complex I** and **Complex II** were similar (Figure 7a). The relation between the complexes and solvent molecules was optically examined at room temperature in seven polar aprotic and protic solvents with varying polarities using UV–vis spectroscopy, and the spectrum of **Complex II** is presented in Figure 7b–d. Due to changes in electron distribution, donor–acceptor interactions resulted in new energy transitions in the spectra. The solutions of **Complex II** were prepared at a concentration of 50 μM by dissolving the complexes in commonly used solvents: DMSO, DMF, isopropyl alcohol (*i*-PrOH), methanol (MeOH), THF, dichloromethane (DCM), and chloroform (CHCl_3_) (Figure 7b).

**Figure 7:**
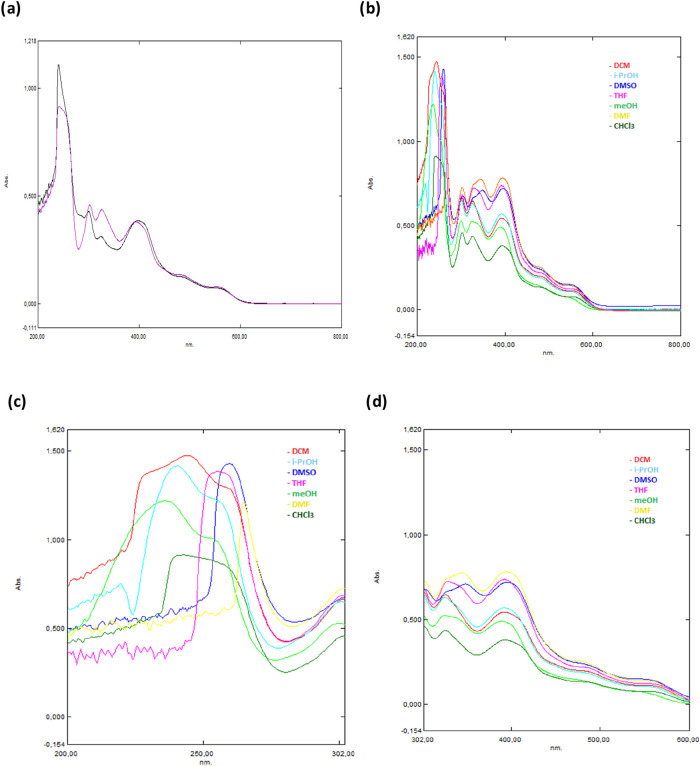
(a) Absorption spectrum of **Complex I** (black color) **and Complex II** (magenta color) in chloroform. (b–d) Absorption spectrum of **Complex II** in different solvents (DMF, DMSO, MeOH, THF, *i*-PrOH, DCM, and CHCl_3_) at 200–800 nm, 215–300 nm, and 302–600 nm, respectively.

Changes in absorption in the ranges of 215–265 and 315–350 nm were particularly noticeable in the spectra, attributed to charge transfer transitions (Figure 7c,d). Additionally, among these solvents, DMSO showed d–d transitions in the range of 600–800 nm with a molar extinction coefficient (ε) of 400 M^–1^cm^–1^.

In the UV–vis spectrum of **Complex II**, π–π* transitions of the phenyl ring were observed at 241.5 nm. Transitions of n–π*, corresponding to the thioamide group, were recorded at 327.50 nm. Charge transfer transitions from the ligand to metal and d–d transitions were observed at 552.50 nm.

The appearance of a new contribution in optical density was observed in the region of 315–350 nm, particularly in the case of the DMSO and DMF solutions. The absorption band in the 315–350 nm region showed a bathochromic shift with increasing solvent polarity and an increase in the optical density. The transition at 348 and 345 nm in spectra belonging to DMSO and DMF solutions, respectively, was observed in decreasing intensity and lower frequency in the spectra of the other solutions. However, this band was not clearly observed in the spectra of CHCl_3_ and CH_2_Cl_2_ solutions.

In addition to the solvent effect, the behavior of **Complex II** with the solvents was investigated at different temperatures and under UV light. For this purpose, the solutions were subjected to temperatures of −10 and 70 °C and irradiated with 366 nm light for 60 min (Figures S10–S17).

The main absorption peak underwent a spectral shift toward shorter wavelengths in the spectra of the other solutions compared to the peak at 264 nm in the aprotic solvent DMF.^104^ The band character of this transition is the same as in the aprotic solvents DMSO and THF, except for the width. These three solvents, hydrogen bond acceptors, have the lowest α value, which means they have the least hydrogen bond donation ability (HBD; Table S2 in the Supporting Information). In other cases, the shift of the main peaks to lower wavelengths is consistent with an increase in the HBD of the solvents.^105^ As a result, it was observed that both NO_2_ and NH_2_ groups on the coligand of the complex structure were able to interact with solvent molecules. The lone pair on the nitrogen atom is able to interact with solvent molecules. Likely, specific solvent–solute interactions through hydrogen bonding have generated the different absorption shifts observed in the spectra of the investigated solvents.^103,106–109^

### 3.5 Electrochemical Studies

Electrochemical characterizations of the starting material and its nickel­(II) complexes (**Complex I** and **Complex II**) were carried out with CV and SWV measurements in DMSO/TBAP electrolyte systems to investigate the redox activity of the starting material and the effects of its coordination to the nickel­(II) cation. As shown in Figure 8a, the starting material illustrates three reduction and two oxidation processes. The reduction processes have very complicated behaviors, which are significantly influenced with the scan vertex potentials. When the vertex potential is returned from −1.25 V, sequential two 1e^–^ imine-based reversible reduction couples at −0.46 V (Red(1)) and at −1.04 V (Red(2)) are observed (Figure 8 and Table 4). When the vertex potential goes through −2.25 V, the third 2e^–^ reduction process (Red(3)) is observed at −2.08 V, which is assigned to the reduction of dianionic imine to the amine product. Due to the irreversibility of the formation of amine products, the previous Red(1) and Red(2) couples become irreversible. Moreover, during the anodic potential scans, two irreversible oxidation waves are also observed at 0.85 V (Oxd.(1)) and 1.15 V (Oxd.(2)).

**Figure 8:**
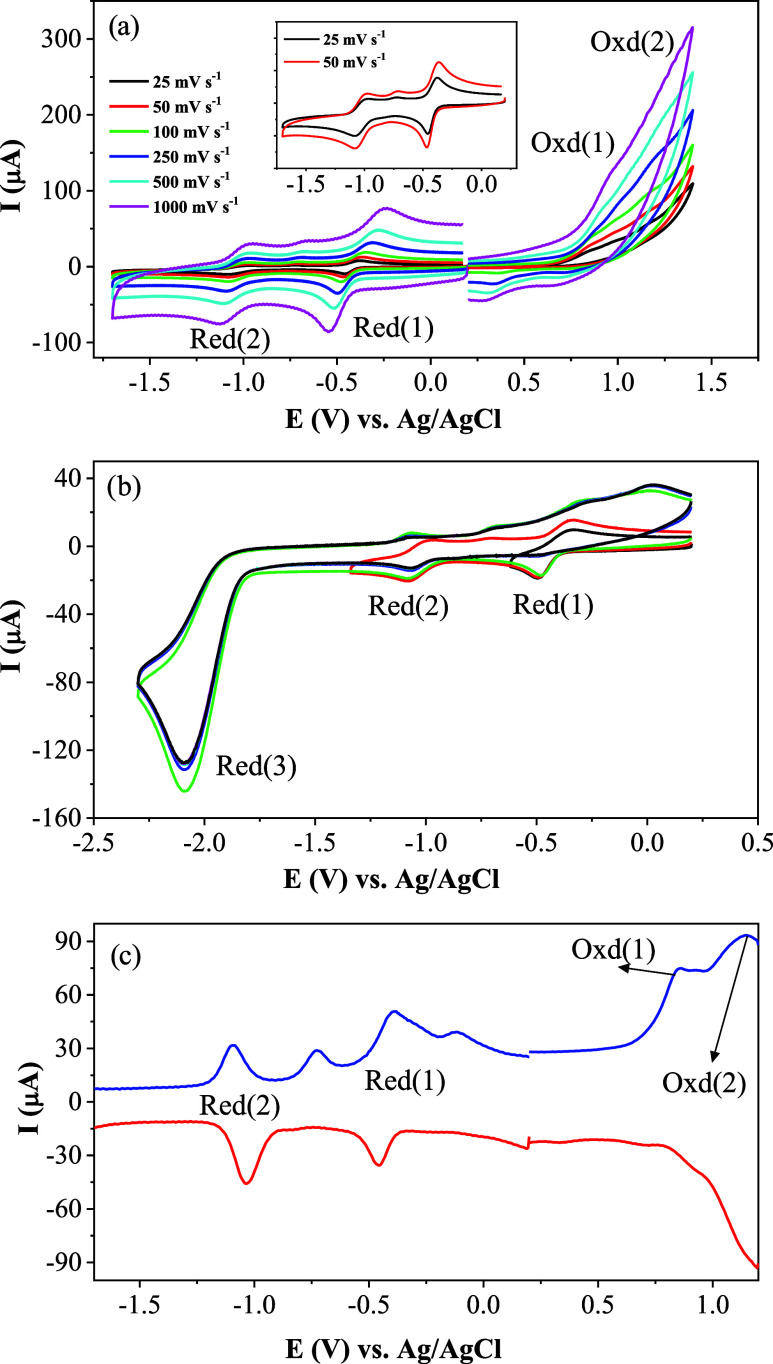
Voltammetric characterizations of the starting material: (a) CV responses at different scan rates, (b) CV responses recorded with different vertex potentials at 100 mV s^–1^ scan rate, and (c) SWV responses at 100 mV s^–1^ scan rate on a GCE electrode in DMSO/TBAP.

**Table 4:** Voltammetric Data for the Complexes

	half-wave potentials of the redox processes (*E*_1/2_)^a^ (V vs Ag/AgCl)		
complexes	ligand oxid.	Ni^II^/Ni^I^	ligand red.
starting material	1.15, 0.85		–0.46, –1.04, –2.08^a^
**Complex I**	1.09, 0.96	–0.92	–1.18, –1.62, –2.00
**Complex II**	1.06, 0.87	–0.97	–1.19, –1.77, –2.02

a *E*_pc_ and *E*_pa_ values were given for the reduction and oxidation processes, respectively.

Coordination of the starting material and the nitro-substituted aniline group to the nickel­(II) cation significantly influenced the reduction responses. As shown in Figures 9 and 10, **Complex I** illustrates similar voltametric behavior to **Complex II** with some slight differences in the reversibility of the redox waves. While **Complex II** gives a reversible Ni^II^/Ni^I^ reduction, this process is observed as completely irreversible with **Complex** **I**, which may be resulted from altering the nitrobenzene moiety of **Complex II** with the dinitrobenzene group on **Complex I**. Except for this difference, all other redox processes of both complexes have similar characteristics.

**Figure 9:**
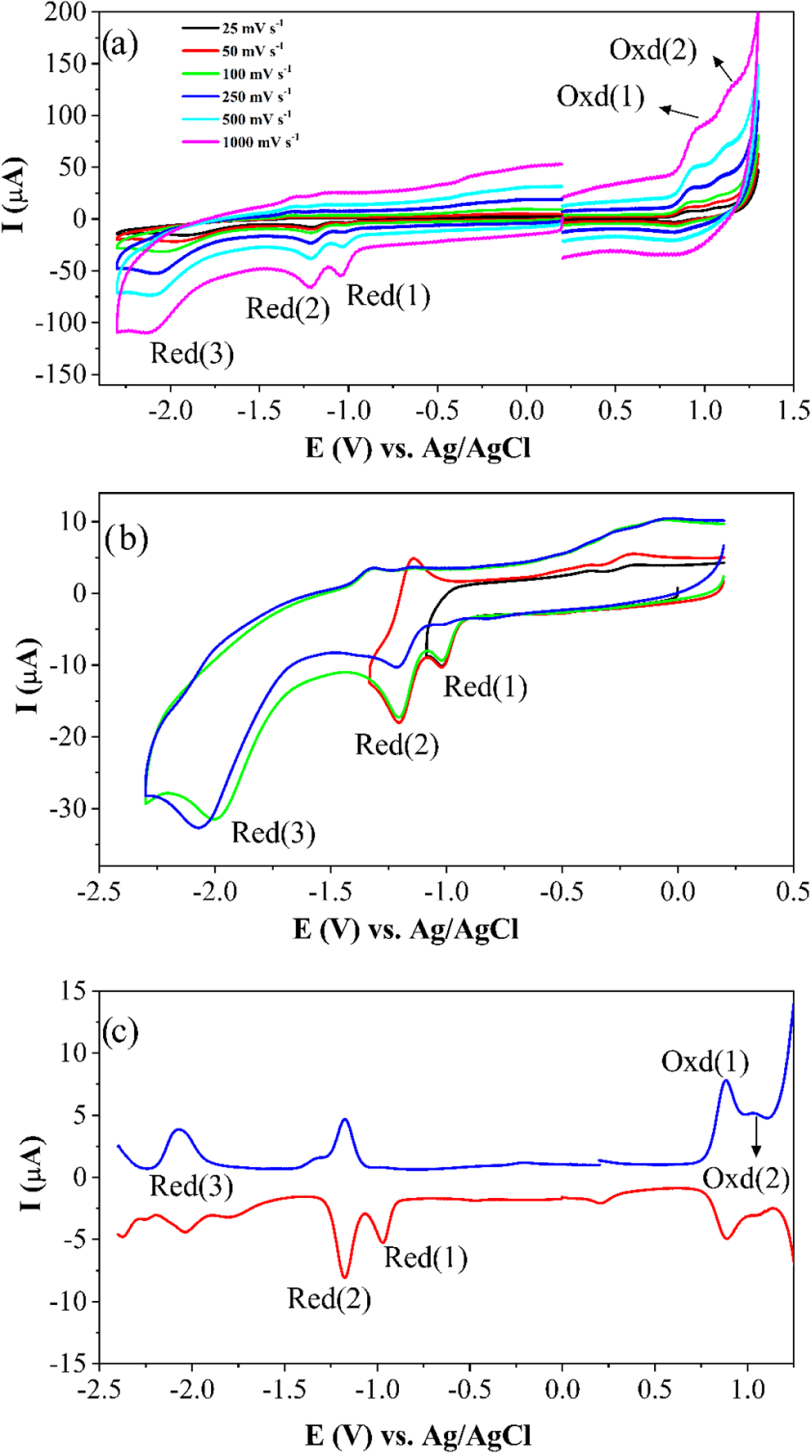
Voltammetric characterizations of **Complex I**: (a) CV responses at different scan rates, (b) CV responses recorded with different vertex potentials at 100 mV s^–1^ scan rate, and (c) SWV responses at 100 mV s^–1^ scan rate on a GCE electrode in DMSO/TBAP.

**Figure 10:**
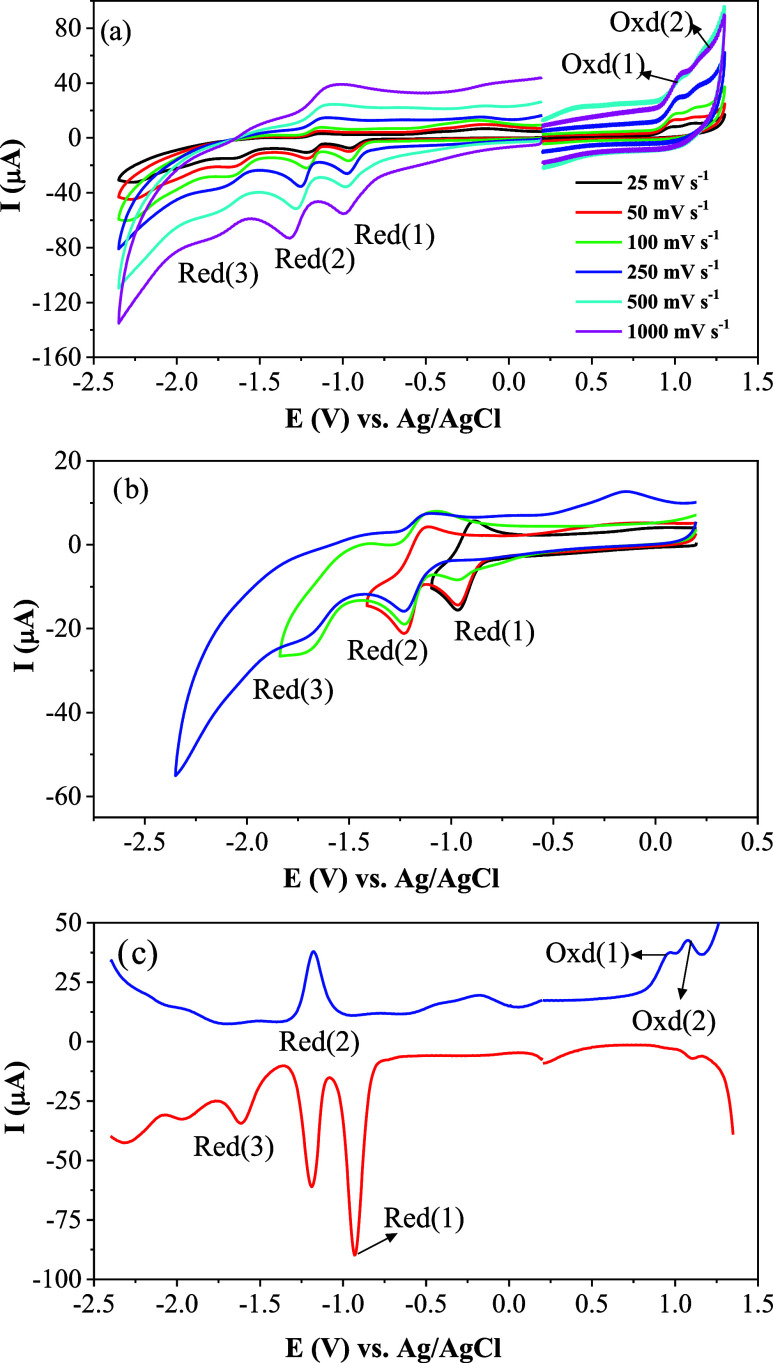
(a) CV responses of **Complex II**, (b) CV responses of **Complex II** recorded with different vertex potentials at 100 mV s^–1^ scan rate, and (c) SWV responses of **Complex II** at 100 mV s^–1^ scan rate on a GCE electrode in DMSO/TBAP.

Figure 10 illustrates the CV and SWV responses of **Complex II**. While the oxidation processes of **Complex II** have similar behavior to those of the starting material, three reduction processes are observed at more negative potentials. When the voltametric and spectroelectrochemical responses (discussed below) are evaluated together, the Red(1) of **Complex II** at −0.92 V can be assigned to Ni^II^/Ni^I^ reduction, and the further reductions are assigned to the reductions of the starting material. Like the starting material, the redox processes of **Complex II** are considerably influenced by the scanned potentials. When the scanned potential is returned just after the second reduction process, both Red(1) at −0.92 V and Red(2) at −1.04 V have electrochemically and chemically reversible character (Figure 10b). However, these processes become chemically irreversible when the vertex potential pass the Red(3) process due to the chemical irreversibility of the amine products formed after the third reduction process. The number of transferred electrons during each redox process was determined with controlled potential electrolysis and found as 1e^–^ transfer for each redox wave.

In situ spectroelectrochemical analyses were carried out to support the mechanism evaluated with voltametric measurements and to record the spectra and color of the electrogenerated species. As shown in Figure 11, the UV–vis spectra of the starting material in DMSO/TBAP show three bands at 270, 305, and 366 nm, which could be assigned to the π–π*, n–σ* transitions of the aromatic ring, phenol, amine, and thioether, and n–π* transition of the azomethine group on the thiosemicarbazone.^110–112^

**Figure 11:**
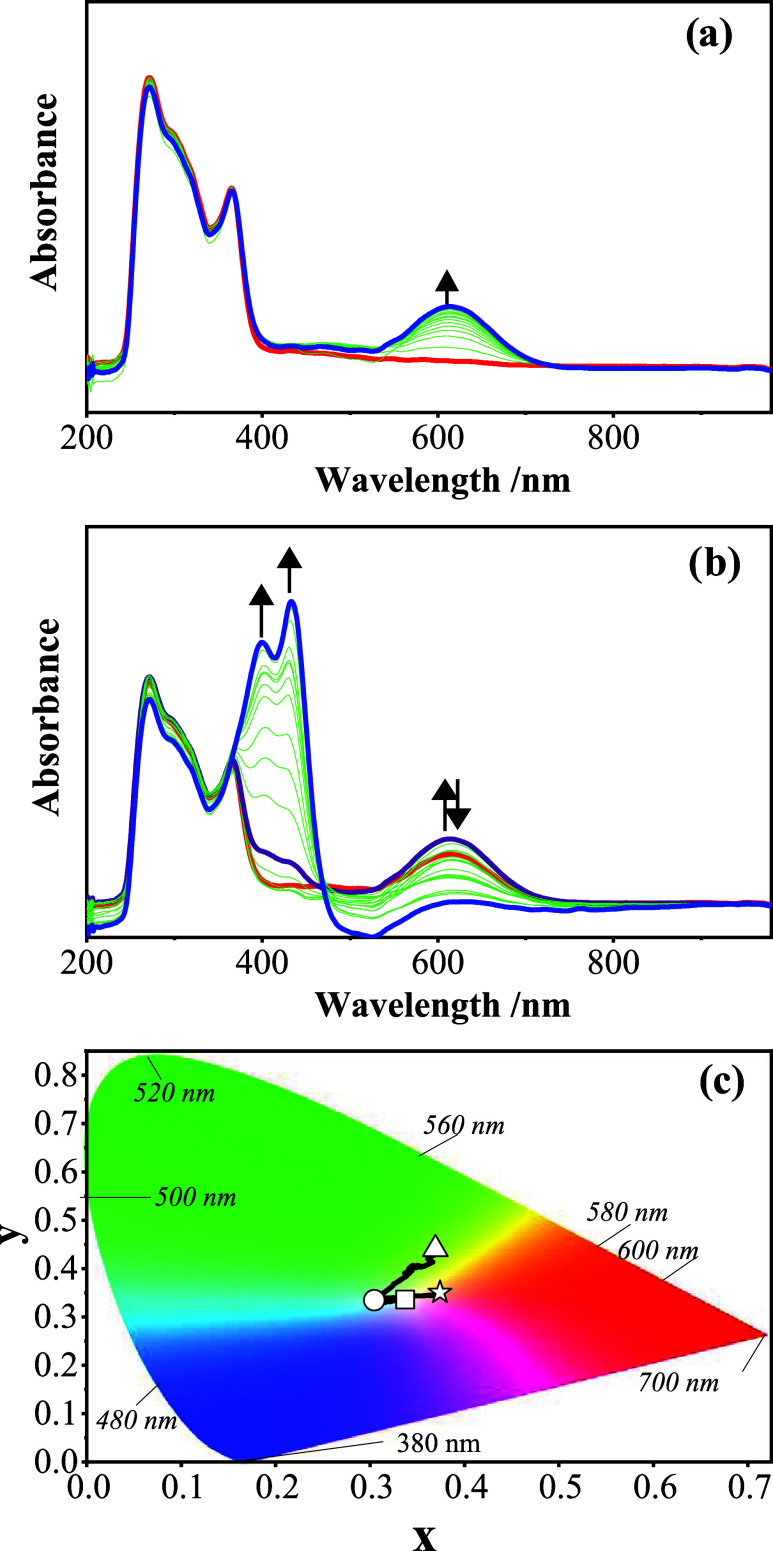
In situ UV–vis spectral changes of starting material observed during the redox reactions in the DMSO/TBAP electrolyte system: (a) *E*_app_ = −0.70 V and (b) *E*_app_ = −1.20 V. (c) Chromaticity diagram (each symbol represents the color of electrogenerated species; □: [L]; ○: [L^]1–^; △: [L]^2–^; ☆: [L]^1+^).

During the first reduction process, while all bands of the starting material remain unchanged, a new band is recorded at 615 nm (Figure 11a). Due to the 1e^–^ imine-based reversible reduction of the starting material, the light-yellow (symbol □; *x* = 0.341; *y* = 0.343) color turns light cyan (symbol ○; *x* = 0.300; *y* = 0.341), as shown in Figure 11d. During the second reduction process, more intense spectral changes are observed, as shown in Figure 11b. While the band at 615 nm decreases in intensity, two new sharp bands are enhanced at 400 and 434 nm. These spectral changes cause a color change from cyan to green (symbol △; *x* = 0.371; *y* = 0.441), as shown in Figure 11d. During the oxidation process, no distinct spectral changes were observed. While all bands remain unchanged, a small to new band was observed at around 500 nm, which causes a color change from light green to orange (symbol ☆; *x* = 0.376; *y* = 0.353).

Both **Complex I** and **Complex II** illustrate almost similar spectral changes; thus, the in situ spectroelectrochemical responses of **Complex II** are given as an example in Figure 12. The UV–vis spectra of **Complex II** in DMSO/TBAP show three bands at 266, 333, and 395 nm, which could be assigned to π–π* transitions of intramolecular charge transfer and n–π* transitions, which belong to the azomethine group. Moreover, the shoulder at 427 nm is observed due to the ligand-to-metal charge transitions. As shown in Figure 12a, a new band at 568 nm with a shoulder at 513 nm is observed while all previous bands remain unchanged during the first reduction reaction. These spectral changes could be assigned to a metal-based electron transfer reaction and support the Ni^II^/Ni^I^ reduction assignment performed with voltametric analyses.

**Figure 12:**
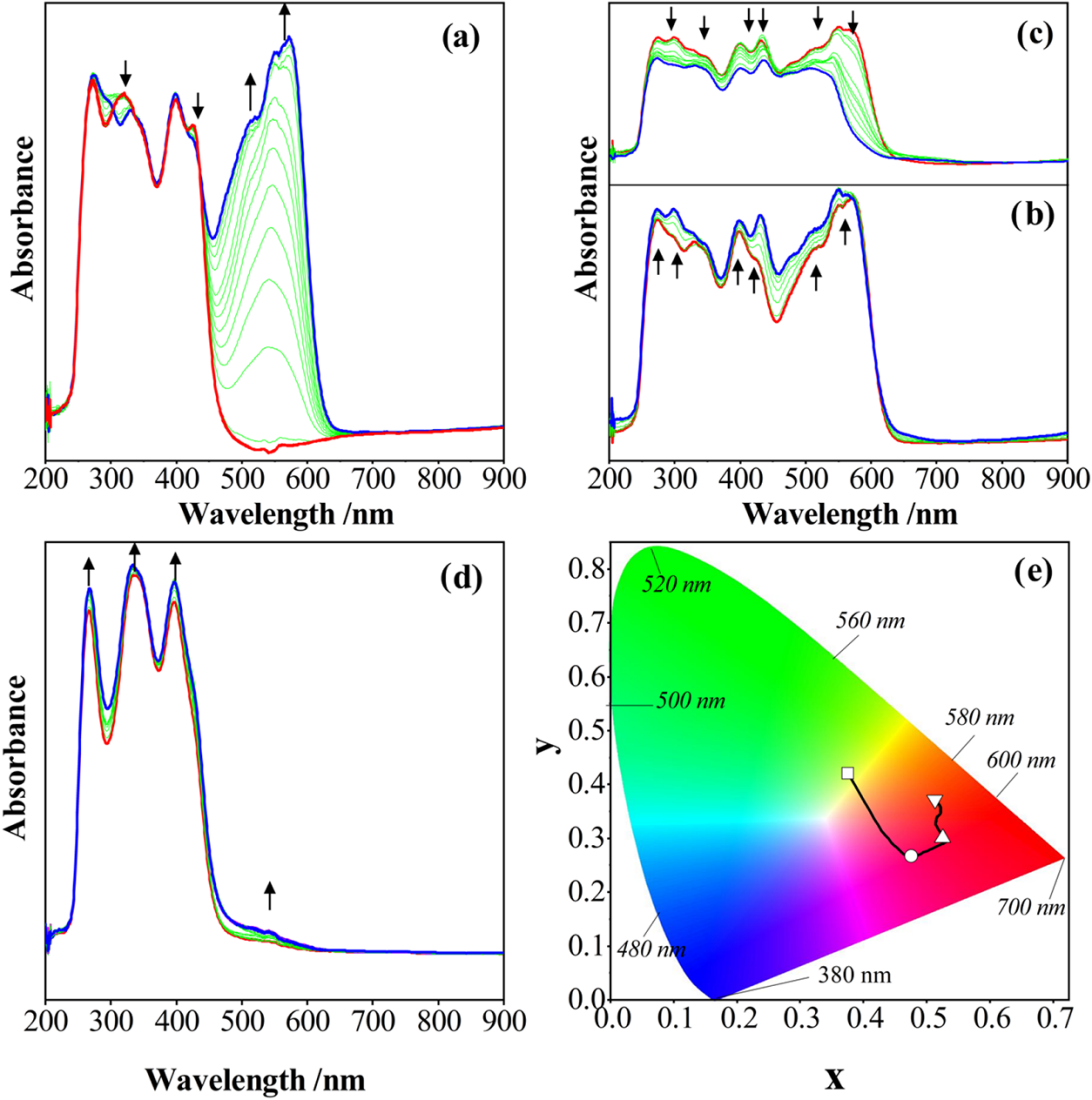
In situ UV–vis spectral changes of **Complex II** observed during the redox reactions in the DMSO/TBAP electrolyte system: (a) *E*_app_ = −1.10 V, (b) *E*_app_ = −1.35 V, (c) *E*_app_ = −2.20 V, and (d) *E*_app_ = 1.25 V. (e) Chromaticity diagram (each symbol represents the color of electrogenerated species; □: [**Ni** ^**II**^ **L]**; ○:[**Ni** ^**I**^ **L** ^**]1**–^; △: [**Ni** ^**I**^ **L** ^**‑1**^ **]** ^**2**–^; ▽: [**Ni** ^**I**^ **L** ^**‑2**^ **]** ^**3**–^; ☆: [**Ni** ^**II**^ **L** ^**1+**^ **]** ^**1+**^).

Both **Complex I** and **Complex II** illustrate almost similar spectral changes; thus, in situ spectroelectrochemical responses of **Complex II** are given as an example in Figure 12. The UV–vis spectra of **Complex II** in DMSO/TBAP show three bands at 266, 333, and 395 nm, which could be assigned to π–π* transitions of intramolecular charge transfer and n–π* transitions, which belong to the azomethine group. Moreover, the shoulder at 427 nm is observed due to the ligand-to-metal charge transitions. As shown in Figure 12a, a new band at 568 nm with a shoulder at 513 nm is observed, while all previous bands remain unchanged during the first reduction reaction. These spectral changes could be assigned to a metal-based electron transfer reaction and support the Ni^II^/Ni^I^ reduction assignment performed with voltametric analyses.

As shown in Figure 12b, slight spectral changes are observed during the ligand-based second and third reduction processes of **Complex II**. Starting material and **Complex II** illustrate similar spectral changes due to the ligand-based characteristics of the oxidation processes observed with all compounds. As shown in Figure 12c, a small increase at around 500 nm is observed with a slight increase in the absorption of the neutral **Complex II** complex. Due to the electron transfer reactions of **Complex II**, greenish color (symbol □; *x* = 0.377; *y* = 0.422) of neutral **Complex II** turns to pinkish red (symbol ○; *x* = 0.470; *y* = 0.268), deep red (symbol △; *x* = 0.524; *y* = 0.297), and reddish orange (symbol ▽; *x* = 0.515; *y* = 0.371) during the reduction reactions (Figure 12d).

## 4 Conclusions

Nickelophilic complexes were synthesized via a one-pot reaction using a salicylaldehyde thiosemicarbazone compound, a nitro-substituted aniline, and nickel­(II) chloride. In the solid state, it was observed that the nickelophilic assemblies were stabilized not only Ni···Ni interactions but also through hydrogen bonding and π–π stacking interactions. During the formation of the supramolecular structure, a template complex with a square-planar geometry was generated from nickel­(II) chloride and salicylaldehyde thiosemicarbazone in the presence of the nitro-substituted aniline compound. The aniline compound, acting as the second coordination ligand, binds to two phenolic oxygen atoms of the template nickel complex via bifurcated hydrogen bonds. Simultaneously, intermolecular π–π interactions occur between the aromatic or chelate rings of two separate template complexes, while also brings the nickel atoms into closer proximity.

The observed solvatochromism in absorption spectra across various solvents indicates solvent-dependent changes in optical properties , highlighting the material’s sensitivity to its chemical environment. Furthermore, the complexes exhibit nonlinear optical behavior, as demonstrated by diffuse reflectance measurements in two distinct phases, suggesting their potential as semiconductor materials. In electrochemical and spectrochemical studies, while the starting material illustrated imine-based reduction processes, these reduction reactions were altered upon coordination with the Ni­(II) cation. Both Ni­(II) complexes illustrated Ni^II^/Ni^I^ reduction processes in addition to the subsequent ligand-centered reductions. The starting material and its complexes illustrated similar oxidation processes, which indicated that the coordination of the starting material to Ni­(II) did not alter its oxidation features. Notably, distinct spectral changes observed during the reduction of both **Complex I** and **Complex II** suggest their potential applicability in various opto-electrochemical applications.

## Supplementary Material







## Notes

List and data of Nickelophines including ONNO coordination mode in the Cambridge Structural Database; table of HBA solvents and their property parameters; infrared spectra of the starting material, **Complex I**, and **Complex II**; UV–vis spectra of the starting material, **Complex I**, and **Complex II**; ^1^H NMR spectra of the starting material, **Complex I**, and **Complex II**; UV spectra of **Complex II** in different solvents; excited/unexcited UV spectrum of **Complex II** in dichloromethane; UV spectrum of **Complex II** in dichloromethane (at room temperature/cold); UV spectrum of **Complex II** in DMSO (at room temperature/cold); UV spectrum of **Complex II** in isopropyl alcohol (at room temperature/cold); UV spectrum of **Complex II** in DMF (at room temperature/cold); UV spectrum of **Complex II** in THF (at room temperature/cold); and UV spectrum of **Complex II** in methanol (at room temperature/cold) (PDF)
Crystallographic data for **Complex I** (CIF)
Crystallographic data for **Complex II** (CIF)
