# Supplementary material for: Thiosemicarbazone Structures Including Nickelophilic Interaction as Well as Both Hydrogen Bonding and π–π Stacking Interactions: NLO, Electrochemical, Chromism, and Spectroelectrochemical Properties
Source: ACS Omega. 2025 Oct 14;10(42):49692–709. doi: 10.1021/acsomega.5c04784 (PMC12573161; doi:10.1021/acsomega.5c04784)
Supplement: Supplementary file 1 [file ao5c04784_si_001.pdf]

Thiosemicarbazone structures including Nickelophilic interaction as well as interactions both hydrogen bonding and π-π stacking: NLO, electrochemical, chromism and spectroelectrochemical properties

Elif Avcu Altıparmak [a], Özlem Uğuz Neli [b,c], Tülay Bal-Demirci [a*], Namık Özdemir [d] and Atıf Koca [c]

[a] *Department of Chemistry, Engineering Faculty, İstanbul University-Cerrahpaşa, 34320, İstanbul, Türkiye*

[b] *Department of Chemistry, Stockholm University, Stockholm, 10691, Sweden*

[c] *Chemical Engineering Department, Engineering Faculty, 34722, Marmara University, Istanbul, Türkiye*

[d] *Department of Physics, Faculty of Science, Ondokuz Mayıs University, 55139, Samsun, Türkiye*

Correspondence: Tulay BAL-DEMİRCİ Department of Chemistry, Engineering Faculty, İstanbul University-Cerrahpaşa, 34320, İstanbul, Türkiye.

E-mail: tulaybal@iuc.edu.tr

Funding information: Istanbul University-Cerrahpaşa

TABLES

Table S1: List of Nickelophines including ONNO coordination mode in Cambridge Structural Database.

Compound	REFCODE	Hydrogen bonding	Ni…Ni (Å)	[Ref]
1	CAPMEO	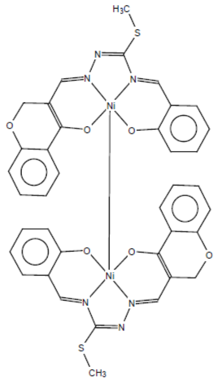	3.408<br>***Space Group No.:*** 9 ***(Å,°)***<br>α 90.00<br>β 115.81(0)<br>γ 90.00	P.Vijayan, P.Anitha, M.Rajeshkumar, P.Viswanathamurthi, P.Sugumar, M.N.Ponnuswamy (2017) *Polyhedron* ,**124,**77
2	DEMDOR	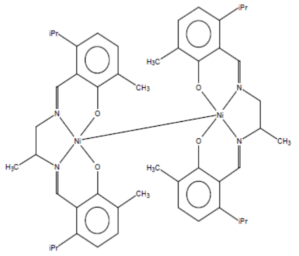	3.314	S.K.Tadavi, A.A.Yadav, R.S.Bendre (2018) *J.Mol.Struct.* ,**1152,** 223
3	FAFSAJ	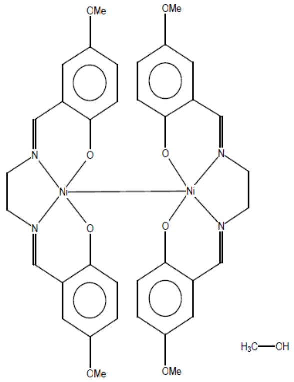	3.414<br>***Space Group No.:*** 14 ***(Å,°)***<br>α 90.00<br>β 92.08(0)<br>γ 90.00	F.Y.Wei (2016) *Koord.Khim.(Russ.)(Coord.Chem.)* ,**42,**44<br>DOI: 10.1134/S1070328416010073

4	FALYIE	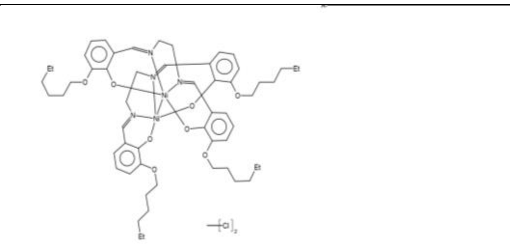	3.281<br><br>Space Group No.: 2 (Å,°) α 97.06(0) β 101.87(0) γ 93.36(0)	D.A.Lukyanov, A.S.Borisova, O.V.Levin (2020) Molbank , 2020,M1174
5	LOKKIG	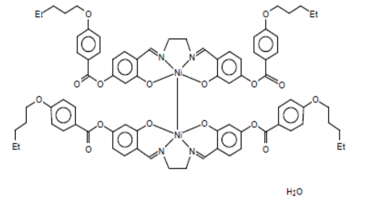	3.336<br><br>***Space Group No.:*** 14 ***(Å,°)***<br>α 90.00<br>β 104.00(0)<br>γ 90.00<br><br>H2O	A.K.Singh, S.Kumari, T.N.G.Row, J.Prakash, K.R.Kumar, B.Sridhar, T.R.Rao (2008) *Polyhedron* ,**27,**3710
6	NOMXIZ	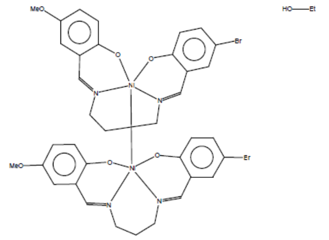	3.363<br><br>***Space Group No.:*** 14 ***(Å,°)***<br>α 90.00<br>β 98.97(0)<br>γ 90.00<br><br>EtOH	L.Rigamonti, A.Forni, S.Righetto, A.Pasini (2019)<br><br>*Dalton Trans.* ,**48,**11217
7<br><br>Bis7(2,2'-(ethane-1,2-diylbis((azanylylidene)methylylidene)) bis(4-<br><br>(phenyldiazenyl)phenolato))-di-nickel dimethyl sulfoxide solvate hydrate	PELVEK	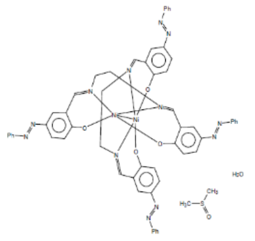	3.263<br><br>***Space Group No.:*** 14 ***(Å,°)***<br>α 90.00<br>β 116.11(0)<br>γ 90.00	A.A.Vereschagin, V.V.Sizov, P.S.Vlasov, E.V.Alekseeva, A.S.Konev, O.V.Levin (2017) *New J.Chem.* ,**41,**13918
8<br><br>bis[2,2'-{ethane-1,2-diylbis[(azanylylidene)methylylidene]} bis[6-<br><br>(trifluoromethoxy)phenolato]]-di-nickel	TITVEA	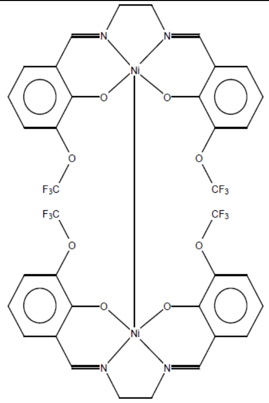	3.295<br><br>Space Group No.: 14 (Å,°) α 90.00 β 99.09(0) γ 90.00	S.Demir Kanmazalp, S.Meral, N.Dege, A.Alaman Agar,<br><br>I.O.Fritsky (2019) *Acta Crystallogr.,Sect.E:Cryst.Commun.* ,**75,**328

9<br><br>bis(μ2-5,11,17,23-tetra-t-butyl-25,27-dihydroxy-26,28-bis(3-(4-(((((2-<br><br>hydroxyphenyl)(oxido)methylidene)amino)imino)methyl)-2-	COTSOV	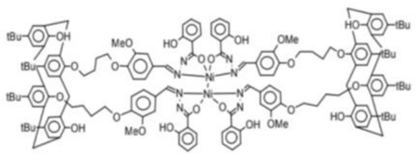	3.251<br><br>Space Group: C2/c Cell: a 31.825(4) b 17.301(2) c 34.465(4)<br><br>Space Group No.: 15 (Å,°) α 90.00 β 99.79(0) γ 90.00	Xin Bi, Jing Sun, Wen-Long Liu, Chao-Guo Yan, *J Incl Phenom Macrocycl Chem* **80**, 235–242 (2014). https://doi.org/10.1007/s10847-014-0382-x
10<br><br>(μ-1-[({2-[({3-[2-({3-[{[4,5-bis(dodecyloxy)-2-{[(2-oxidonaphthalen-1-yl) methylidene]amino}phenyl]imino}methyl]-5-t-butyl-2-oxidophenyl}methyl)<br><br>prop-2-en-1-yl]-5-t-butyl-2-oxidophenyl}methylidene)amino]-4,5-bis(dodecyloxy)phenyl}imino)methyl]naphthalen-2-olato)-di-nickel<br><br>ethanol tetrahydrofuran solvate	GIKVAA	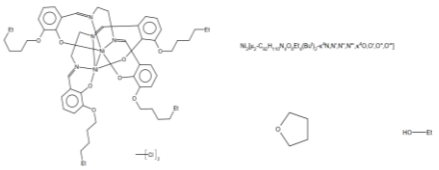	3.290<br><br>Space Group: P-1 Cell: a 13.602(11) b 20.351(14) c 21.939(18) Space Group No.: 2 (Å,°) α 69.47(2) β 89.09(3) γ 78.50(3)	H.Achira, Isao Yoshikawa, H.Houjou (2018) Thermochim.Acta ,669,52
11	GOQMAD	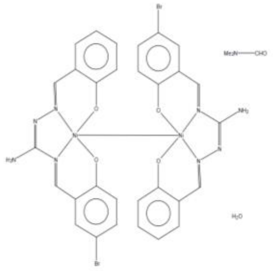	3.419	Olga Yu. Vassilyeva, Elena A. Buvaylo, Vladimir N. Kokozay, Sergey L. Studzinsky, Brian W. Skelton, Georgii S. Vasyliev, Acta Crystallographica Section E: Crystallographic Communications<br><br>Volume 78| Part 2| February 2022| Pages 173-178

Table S2. Hydrogen Bond Acceptor (HBA) Solvents and their property parameters

	α	β	π*	$E_T(30)$	*DN*	*AN*	Acity	Basity	Z	Z'
Methanol	98	66	60	55.4	30.0	41.3	75	50	83.6	79.4
Chloroform	20	10	58	39.1	4.0	23.1	42	73	63.2	57.8
dichloromethane	13	10	82	40.7	1.0	20.4	33	80	64.7	59.3
Isopropanol, *i*-PrOH	76	84	48	49.2	36.0	33.5	59	44	76.3	72.4
Dimethylformamide,DMF	00	69	88	43.8	26.6	16.0	30	93	68.4	65.3
Dimethyl sulfoxide,DMSO	00	76	1 00	45.1	29.8	19.3	34	1.08	70.2	67.0
Tetrahydrofuran,THF	00	55	58	37.4	20.0	8.0	17	67	58.8	56.0

HBD ability α, HBA ability β, polarity/polarizability π, Reichardt empirical polarity $E_T(30)$, donor number DN, acceptor number AN, Acity, Basity, polarity Z, polarity Z' (Y. Marcus, Chemical Society Reviews, 1993, 409-416)*

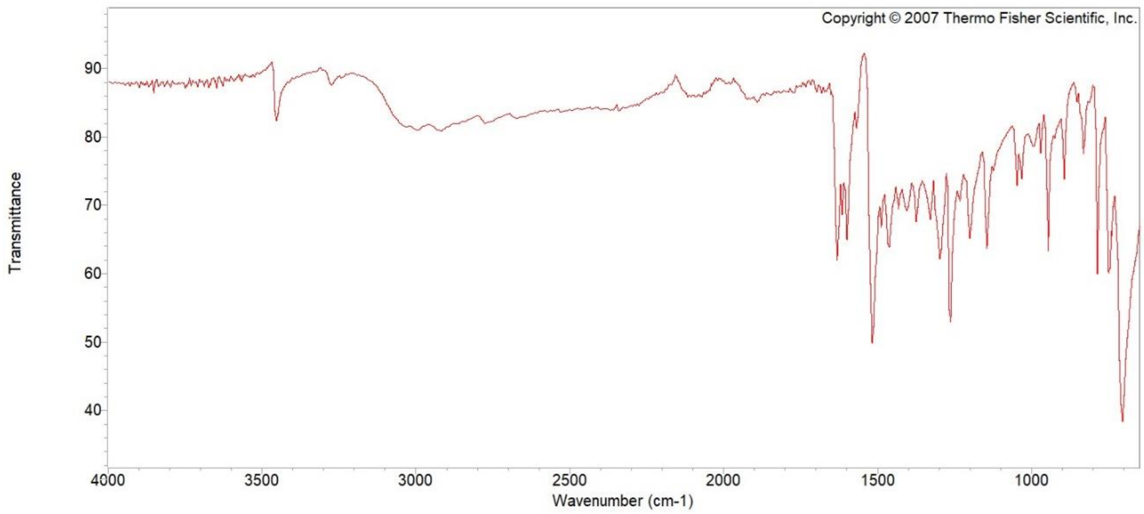

Figure S1: Infrared Spectrum of the Starting Material.

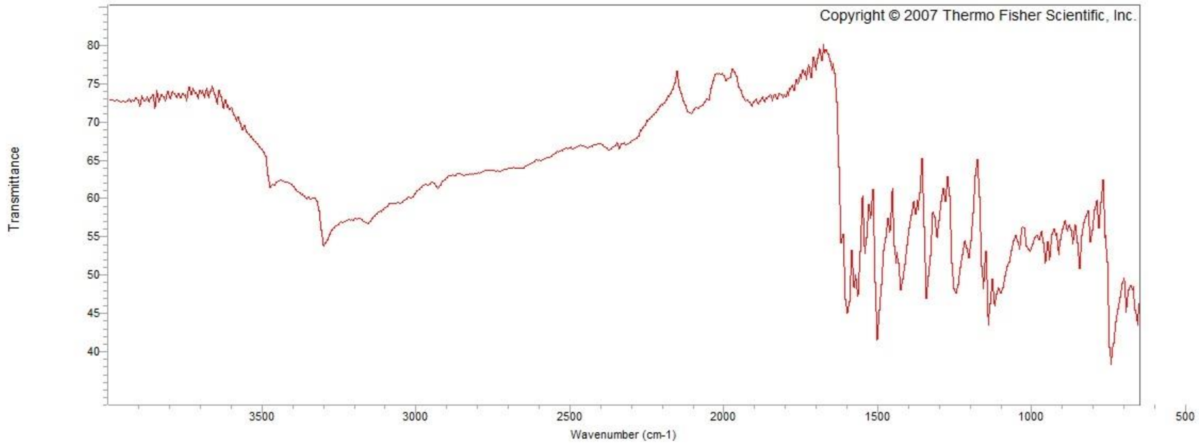

Figure S2: Infrared Spectrum of the **Complex I.**

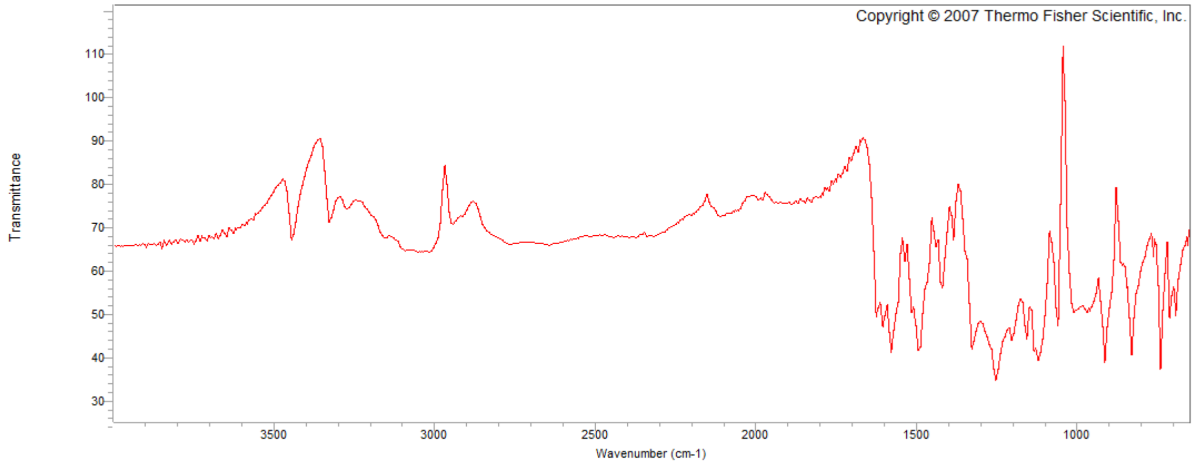

Figure S3: Infrared Spectrum of the **Complex II.**

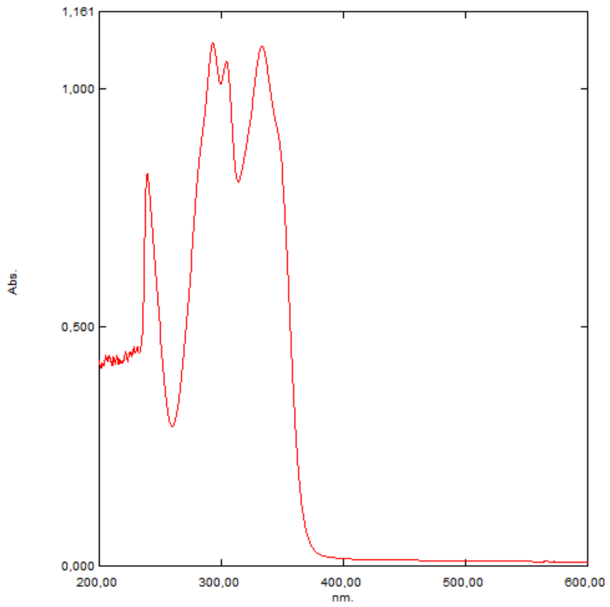

Figure S4: UV-Vis Spectrum of the Starting Material.

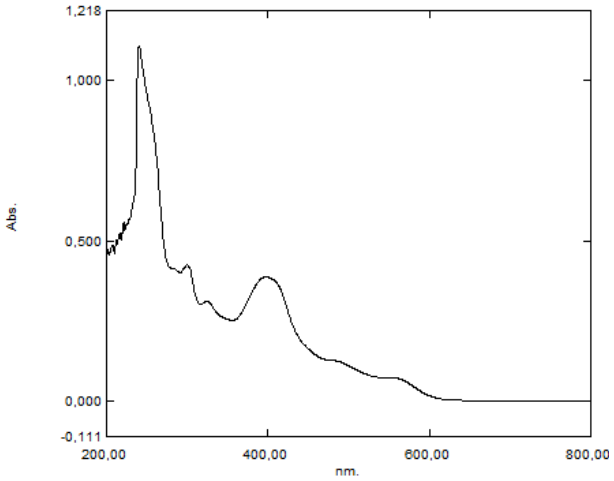

Figure S5: UV-Vis Spectrum of the **Complex I.**

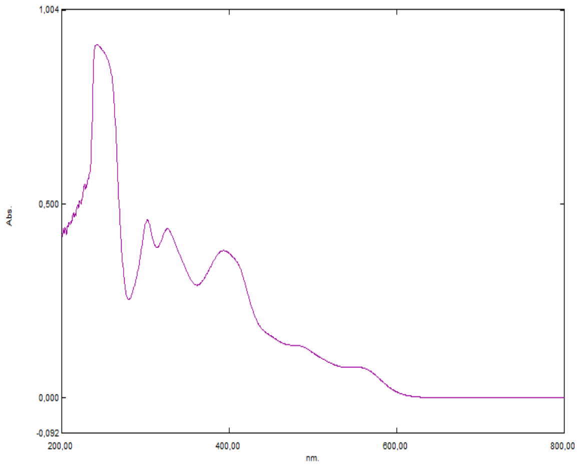

Figure S6: UV-Vis Spectrum of the **Complex II**

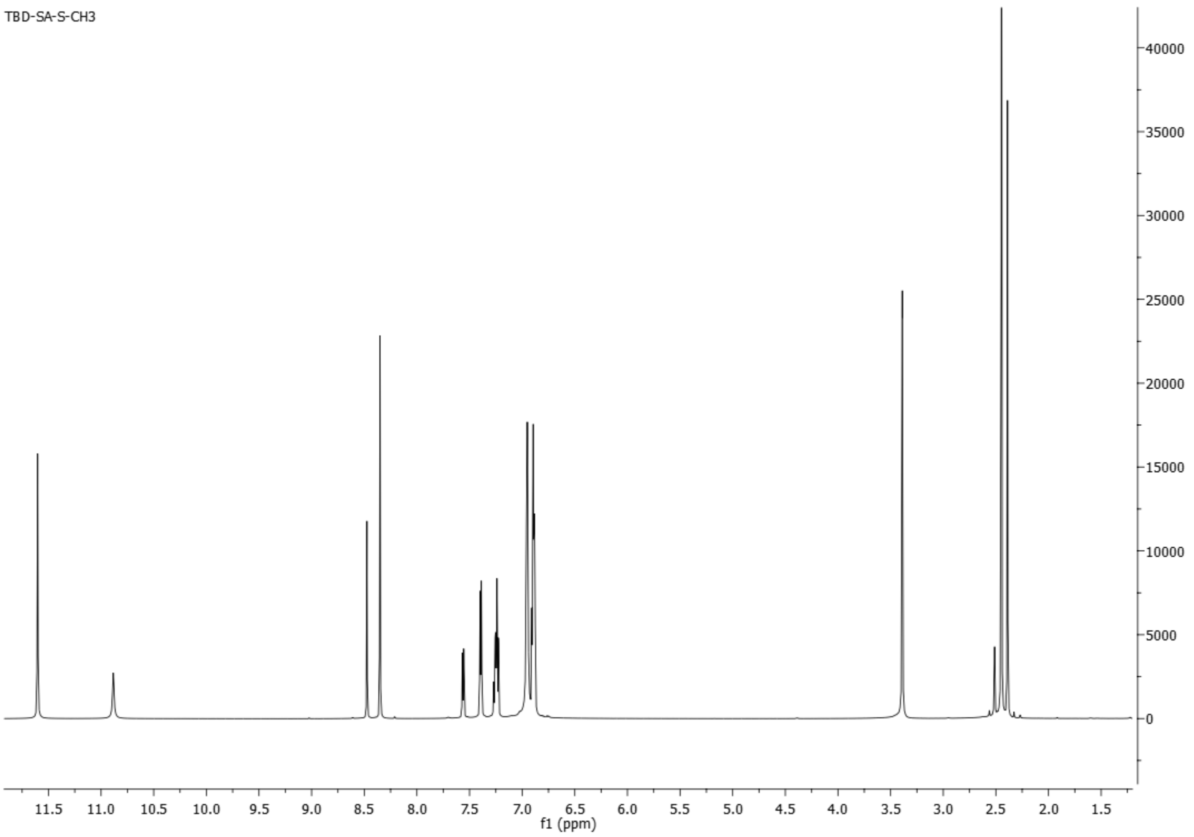

Figure S7: ^{1}H-NMR Spectrum of the Starting Material.

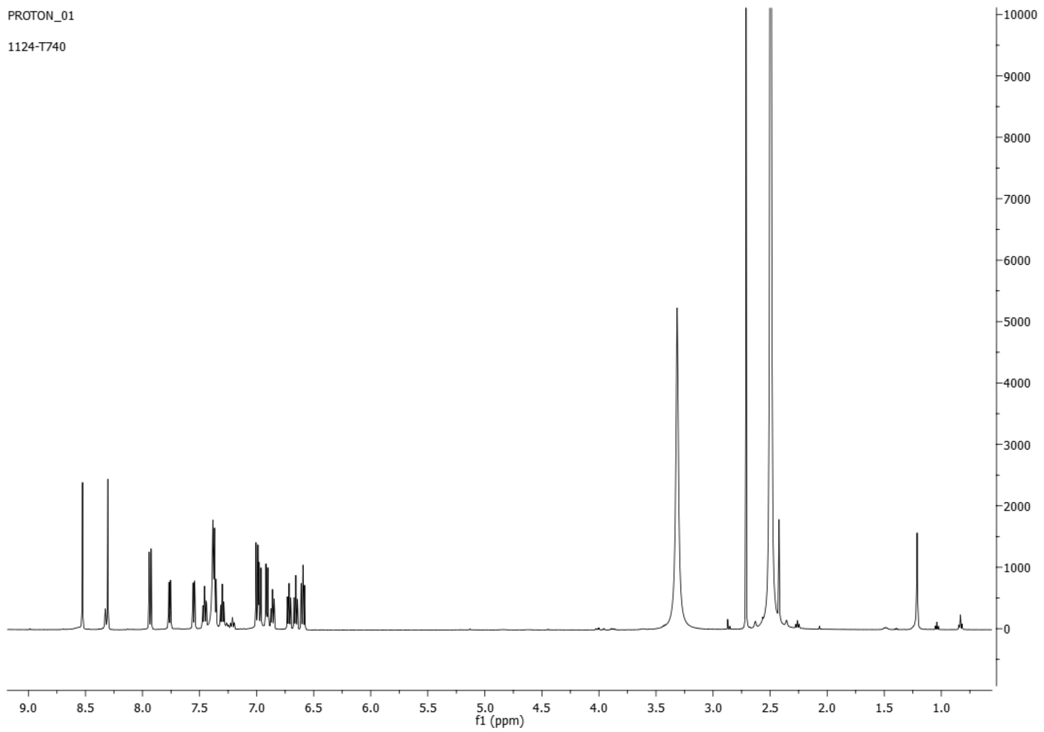

Figure S8: ^{1}H-NMR Spectrum of the **Complex I.**

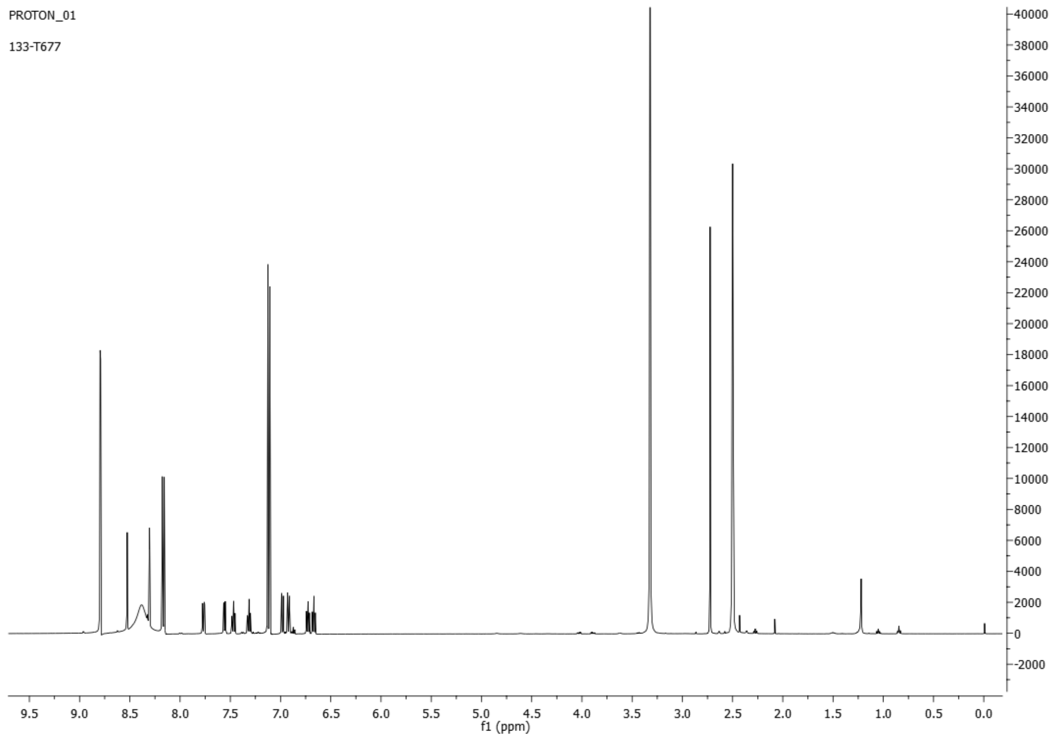

Figure S9: ^{1}H-NMR Spectrum of the **Complex II.**

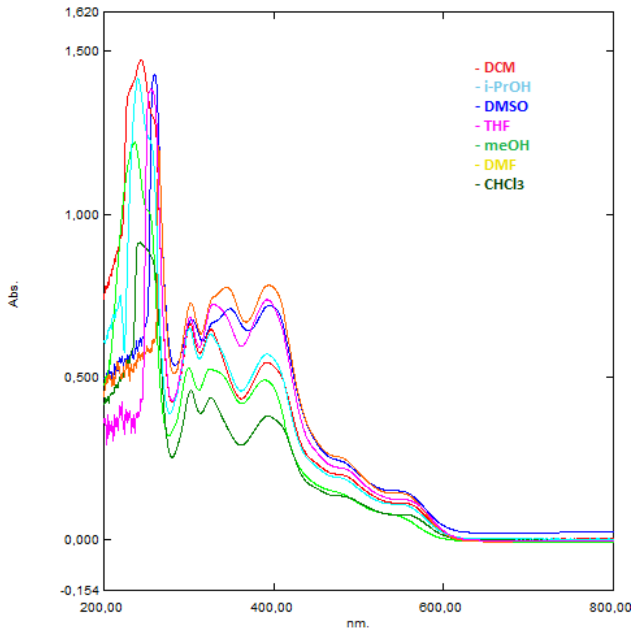

Figure S10: The UV spectrum of **Complex II** in different solvents.

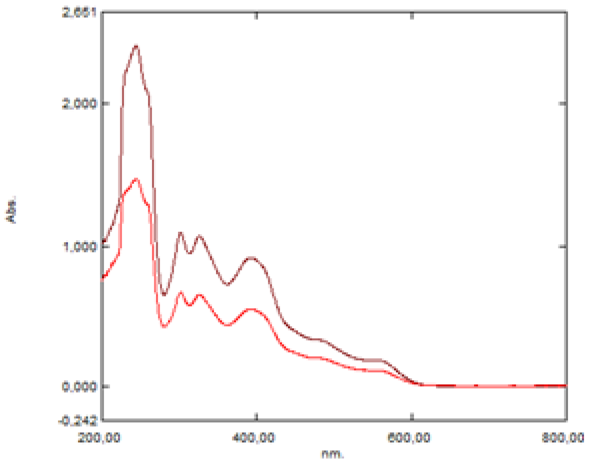

Figure S11: UV spectrum of **Complex II** in dichloromethane (The excited/unexcited, (at 366 nm, 1h).

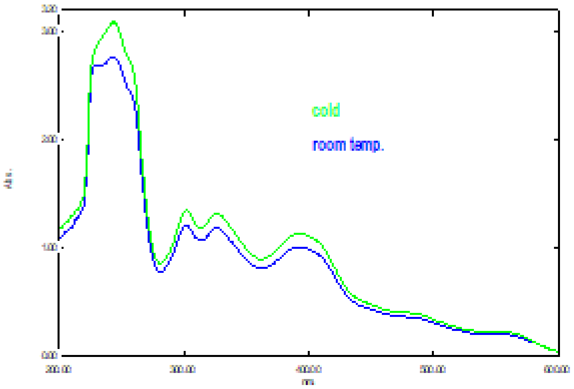

Figure S12: UV spectrum of **Complex II** in dichloromethane (at the room temperature/cold).

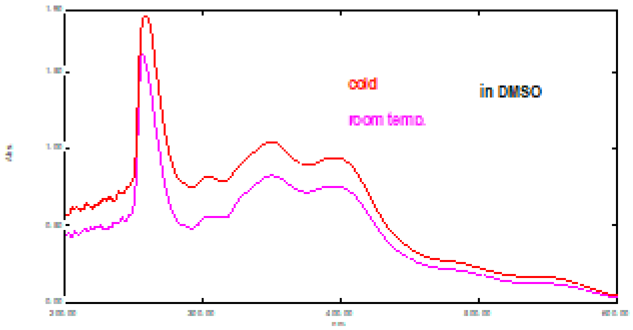

Figure S13: UV spectrum of **Complex II** in DMSO (at the room temperature/cold).

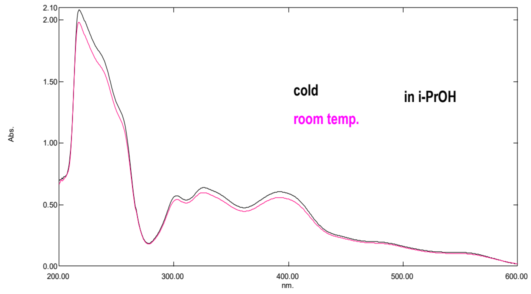

Figure S14: UV spectrum of **Complex II** in isopropyl alcohol (at the room temperature/cold).

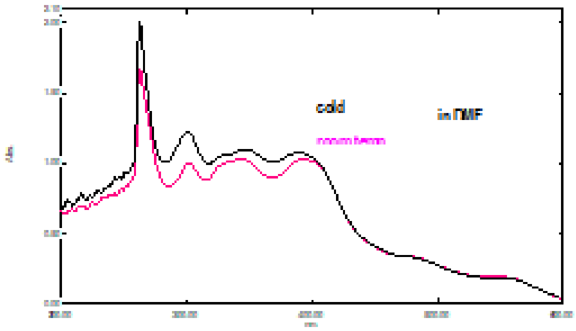

Figure S15: UV spectrum of **Complex II** in DMF (at the room temperature/cold).

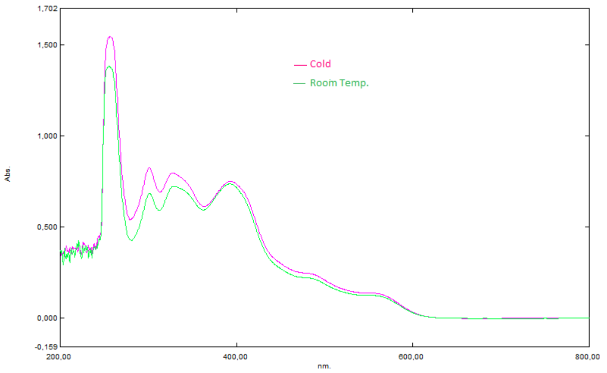

Figure S16: UV spectrum of **Complex II** in THF (at the room temperature/cold).

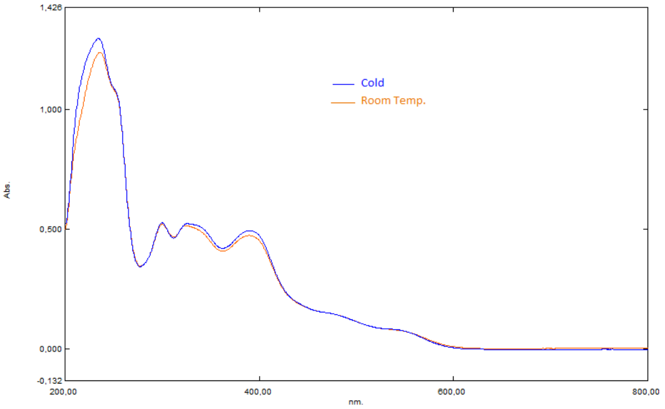

Figure S17: UV spectrum of **Complex II** in methanol (at the room temperature/cold).
